# Root parasitic plant *Orobanche aegyptiaca* and shoot parasitic plant *Cuscuta australis* obtained Brassicaceae-specific *strictosidine synthase-like* genes by horizontal gene transfer

**DOI:** 10.1186/1471-2229-14-19

**Published:** 2014-01-13

**Authors:** Dale Zhang, Jinfeng Qi, Jipei Yue, Jinling Huang, Ting Sun, Suoping Li, Jian-Fan Wen, Christian Hettenhausen, Jinsong Wu, Lei Wang, Huifu Zhuang, Jianqiang Wu, Guiling Sun

**Affiliations:** 1 Key Laboratory of Economic Plants and Biotechnology, Kunming Institute of Botany, Chinese Academy of Sciences, 132 Lanhei Road, Heilongtan, Kunming 650201, Yunnan, China; 2 College of Life Science, Henan University, 85 Minglun Street, Kaifeng 475001, Henan, China; 3 Key Laboratory of Biodiversity and Biogeography, Kunming Institute of Botany, Chinese Academy of Sciences, Kunming 650201, China; 4 Department of Biology, East Carolina University, Greenville, NC 27858, USA; 5 State Key Laboratory of Genetic Resources and Evolution, Kunming Institute of Zoology, Chinese Academy of Sciences, 32 Jiaochang East Road, Kunming 650223, Yunnan, China

**Keywords:** *Cuscuta*, Horizontal gene transfer, New genes, *Orobanche*, Parasitic plants, Strictosidine Synthase-Like Genes

## Abstract

**Background:**

Besides gene duplication and *de novo* gene generation, horizontal gene transfer (HGT) is another important way of acquiring new genes. HGT may endow the recipients with novel phenotypic traits that are important for species evolution and adaption to new ecological niches. Parasitic systems expectedly allow the occurrence of HGT at relatively high frequencies due to their long-term physical contact. In plants, a number of HGT events have been reported between the organelles of parasites and the hosts, but HGT between host and parasite nuclear genomes has rarely been found.

**Results:**

A thorough transcriptome screening revealed that a *strictosidine synthase-like* (*SSL*) gene in the root parasitic plant *Orobanche aegyptiaca* and the shoot parasitic plant *Cuscuta australis* showed much higher sequence similarities with those in Brassicaceae than with those in their close relatives, suggesting independent gene horizontal transfer events from Brassicaceae to these parasites. These findings were strongly supported by phylogenetic analysis and their identical unique amino acid residues and deletions. Intriguingly, the nucleus-located *SSL* genes in Brassicaceae belonged to a new member of *SSL* gene family, which were originated from gene duplication. The presence of introns indicated that the transfer occurred directly by DNA integration in both parasites. Furthermore, positive selection was detected in the foreign *SSL* gene in *O. aegyptiaca* but not in *C. australis*. The expression of the foreign *SSL* genes in these two parasitic plants was detected in multiple development stages and tissues, and the foreign *SSL* gene was induced after wounding treatment in *C. australis* stems. These data imply that the foreign genes may still retain certain functions in the recipient species.

**Conclusions:**

Our study strongly supports that parasitic plants can gain novel nuclear genes from distantly related host species by HGT and the foreign genes may execute certain functions in the new hosts.

## Background

New genes provide novel traits to organisms and thus contribute to the adaption of species to new ecological niches [1,2]. The mechanisms by which organisms acquire new genes have been intensively studied and the rapidly accumulating genomic data have further supported the idea that most genes were originated from duplication events [3,4]. Besides gene duplication, retroposition, exon shuffling, trans-splicing, and *de novo* gene evolution, horizontal gene transfer (HGT) represents another critical source of gaining novel genes by directly introducing new genes to distantly related recipient species [1].

A large number of HGT events have been recognized in prokaryotes [2] and unicellular eukaryotes [5-7]. Although in multicellular animals, the separation of germ line cells from the soma expectedly leads to low HGT frequencies, genes of prokaryotic, fungal or plant origins have been discovered in diverse animals including bdelloid rotifer [8,9], tunicates [10], jelly fishes [11], starlet sea anemones [12], nematodes [13], aphids [14,15] and other insects [16,17]. The evolutionary significance of HGT has been well illustrated in some animals. For instance, acquiring two carotenoid biosynthetic genes from fungi endowed the pea aphids with red body color, based on which the variation between green and red aphids is further driven by the predators and parasites [18].

HGT is also involved in the adaptation and genome evolution of plants [19,20]. In nonvascular plants, Yue et al. identified 57 gene families in a moss nuclear genome with prokaryotic, fungal, and viral origins [21]. Most of these genes were most likely transferred to the ancestor of green plants and may have played significant roles during the transition of plants from being aquatic to terrestrial [21]. Extensive HGT has occurred between the organellar genomes of higher plants [19,22-24]. However, gene transfer between plant nuclear genomes was rarely reported [19,22]. Some exceptions are that a Mu-like element (MULE) in higher plants was found to be transferred between rice and *Setaria* nuclear genomes [25], and similarly, four independent genes in the C4 photosynthesis pathway were recurrently transferred from C4 to C3 plants in Panicoideae, and such HGT events may explain the origin of C4 plants in different species [26]. These HGTs were all between nuclear genomes and it is still unclear how these HGT events happened, since there are no direct physical interactions between donors and recipients.

Physical contact between the donors and the recipients theoretically facilitate HGT occurrence, since it increases the chance of genetic material transfer [7,27]. Parasites and hosts can form long-lasting intimate physical contact and thus parasitism may result in relatively high frequencies of HGT [5,7]. Consistent with this scenario, the majority of HGT events in higher plants found so far are from plant parasitic systems. In plants, hundreds of host organelle genes have been reported to be transferred from hosts to parasites [23,28,29], or vice versa [30-32], with most being targeted to the recipient mitochondria. In contrast, only few cases of HGT between the nuclear genomes of parasitic plants and hosts were reported [19,22]. The first confirmed HGT in parasitic systems involving nuclear genomes was that a gene with unknown function was transferred from a monocot host (likely *Sorghum*) to the eudicot parasite plant *Striga hermonthica*[33], and this was detected by analyzing the expressed sequence tags of *S. hermonthica*. With the rapid development of the next-generation sequencing technologies, increasing amount of transcriptome data become available. *Rafflesia cantleyi* (Rafflesiaceae) is an obligate and holoparasitic plant, and by screening the transcriptome data of this parasite and its obligate host *Tetrastigma rafflesiae,* Xi et al. proposed that several dozen genes were transferred from the host to the parasite, with most probably encoded by the nuclear genomes [34]. Likewise, Zhang et al. found that an albumin gene, encoding a seed storage and insect toxin protein, was transferred from legumes to the root parasitic plant *Orobanche aegyptiaca* and shoot parasitic plant *Cuscuta pentagona* and probably retained the same function after HGT [35].

Plants in the genus *Orobanche* (Orobanchaceae) are root parasites and form one of the largest groups of holoparasitic plants. Most *Orobanche* species have a narrow host range, while *O. aegyptiaca* is one of the exceptions, as it parasitizes many plants including Brassicaceae, Leguminosae, Solanaceae, Apiaceae, Asteraceae, and Cucurbitaceae [35,36]. *O. aegyptiaca* is mainly distributed in Mediterranean region and western Asia and can also been found sporadically in Africa, Australia, America, and eastern Asia [37]. *Cuscuta* (Convolvulaceae) plants are shoot parasites and represent another group of obligate parasitic plants, and most members of this genus have broad host ranges, which greatly overlap with the plant families infected by *O. aegyptiaca*[38], and have a wide geographic distribution that is similar to that of *O. aegyptiaca*[37].

The inconsistence between a species tree and a gene tree is an important indication of the occurrence of HGT. The genome data of *Mimulus guttatus* and *Solanum* (*S. tuberosum* and *S. lycopersicum*), the ordinal relatives of *Orobanche* and *Cuscuta* respectively, have been released [39]. *M. guttatus* (Phrymaceae) and *O. aegyptiaca* belong to the order Lamiales, and *Solanum* spp. (Solanaceae) and *C. australis* (Convolvulaceae) belong to the order Solanales. The available genomic information from *M. guttatus* and *Solanum* has provided an excellent opportunity for identifying HGT in *Orobanche* and *Cuscuta*. Here, we report that *strictosidine synthase*-*like* (*SSL*) genes from Brassicaceae were co-opted by the two parasitic plants, *O. aegyptiaca* and *C. australis*. Furthermore, these *SSL* genes belong to a new member of the *SSL* gene family and were originated by gene duplication uniquely in Brassicaceae. The presence of introns in the foreign *SSLs* of parasitic plants strongly suggests that host DNA, but not mRNA, was directly integrated into *O. aegyptiaca* and *C. australis* respectively. Furthermore, the expression levels of the *SSL* genes in *O. aegyptiaca* and *C. australis* varied in different developmental stages and organs and the *SSL* gene in *C. australis* was inducible after wounding. These results support the scenario that during parasitization nuclear genes can be transferred from hosts to parasitic plants, and the foreign genes may provide their new hosts with novel traits, which might be beneficial for adaptation.

## Results

### Identification of foreign *SSL* genes in *Orobanche aegyptiaca* and *Cuscuta australis*

*O. aegyptiaca* is a root holoparasitic plant that parasitizes many plant species. Recently, a large set of transcriptome data was released (Parasitic Plant Genome Project; [40]). Taking advantage of these published databases, we downloaded the assembled transcriptomes of *O. aegyptiaca* and screened for foreign genes (see Additional file 1 for the procedure). More than 2100 sequences were obtained initially which were predicted as HGT candidates by AlienG [41]. Among these, a 691-bp transcript fragment showed 88 and 89% identity at the nucleotide and amino acid level, respectively, to a *strictosidine synthase-like* (*SSL*) gene in *Arabidopsis thaliana* (hereafter, Arabidopsis) (AT2G41300), while it was highly divergent from the homologs in *Orobanche* relative *Mimulus guttatus* (49% identity at the amino acid level and no significant similarities at the nucleotide level). To obtain the complete *SSL* transcript sequence, we re-assembled the RNA-seq datasets of *O. aegyptiaca* using Trinity [42] and obtained a 1597-bp cDNA sequence with a putatively complete open reading frame (ORF) encoding 369 amino acids (hereafter *OaSSL*, NCBI accession number: KF817594).

Considering that the shoot parasitic plant *C. australis* also parasitizes Brassicaceous plants, we investigated whether *C. australis* also gained the *SSL* gene. A homemade transcriptome assembly of *C. australis* was searched using *OaSSL* as the query, and 2 highly similar transcripts were found, which were very likely derived from one gene by alternative splicing (see below). This *C. australis SSL* (hereafter *CaSSL*) also showed high similarities with the same Arabidopsis *SSL* at the nucleotide and amino acid level (84 and 88%, respectively), but exhibited much lower similarities with its Solanales homologs in *S. tuberosum* and *S. lycopersicum* (49% and 50% at the amino acid level, no significant similarities at the nucleotide level). These data strongly suggested that these two parasitic plants acquired a *SSL* gene from Arabidopsis or certain other Brassicaceous species.

**Figure 1:**
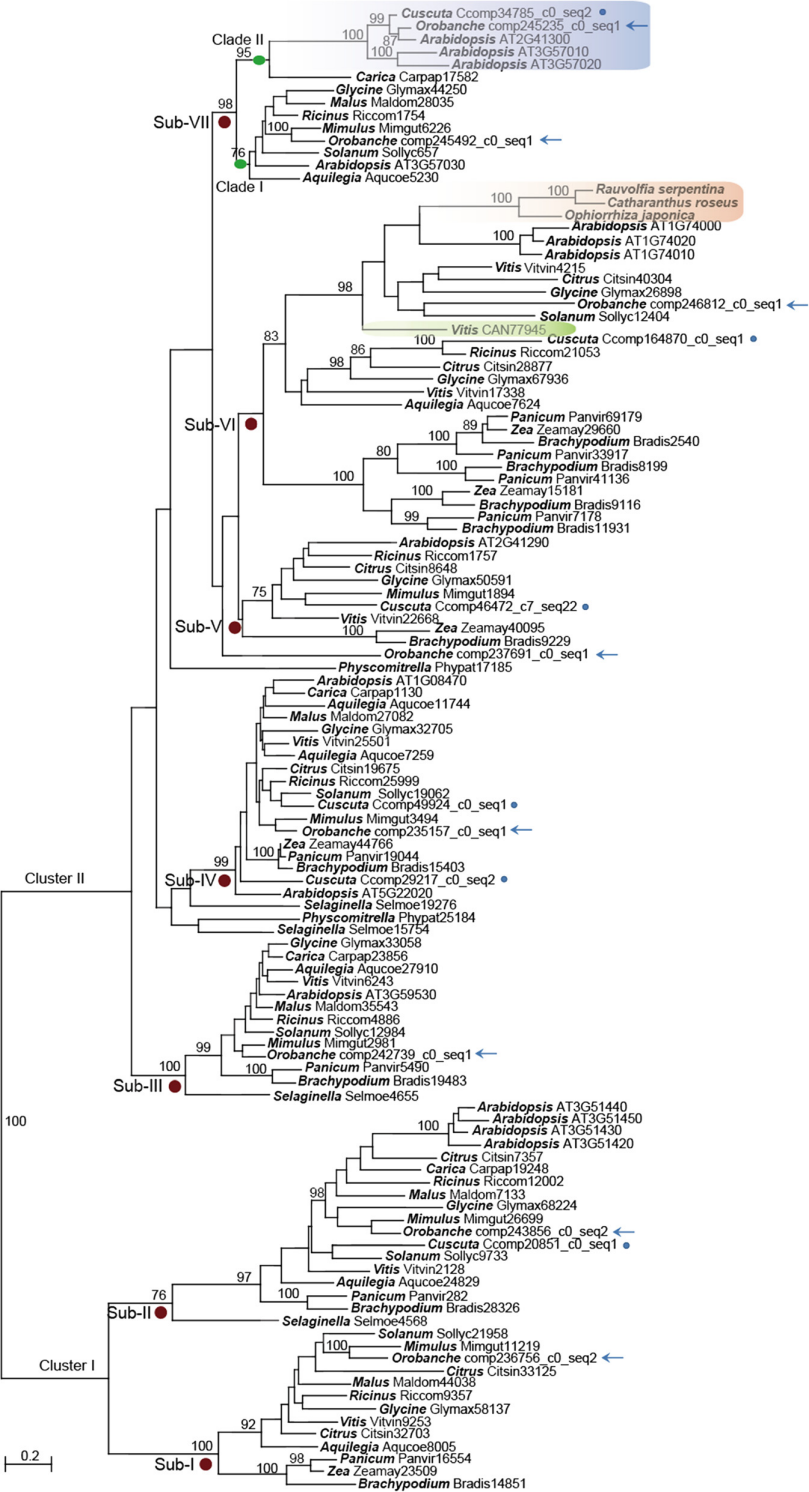
**Molecular phylogeny of the strictosidine synthase-like (SSL) protein family in plants.** To reduce the size of the tree, only one sequence among multiple highly similar copies in each plant was used, except that all the SSLs of *Arabidopsis thaliana* were used. Genus names and sequence IDs are shown in the nodes. Numbers above branches indicate bootstrap support values of maximum likelihood when they are more than 75%. Red circles below the branches indicate Sub-families I to VII (Sub-I to Sub-VII). Green circles indicate the branches of Clade I and (Brassicales-specific genes). The sequences from parasitic plants *O. aegyptiaca* and *C. australis* are indicated by arrows and dots, respectively. The interested *SSL* genes in *O. aegyptiaca* and *C. australis* and their Brassicaceae donor are indicated in blue. Three sequences from *Rauvolfia serpenti*, *Catharanthus roseus*, and *Ophiorrhiza japonica* with biochemical evidence for the activity of strictosidine synthase are indicated in orange. SSL in *Vitis vinifera*, which has no strictosidine synthase activity detected, is indicated in green.

To gain insight into the evolution of *SSL* genes and rule out the possibility that *OaSSL* and *CaSSL* were originated from non-Brassicaceous plants, *SSL* sequences from 15 representative plant genomes and the two parasitic plants were used for constructing a gene tree (Figure 1). Phylogenetic analysis using all *SSL* members in Arabidopsis and the representative homolog sequences from other species (the sequences that clustered as sister branches in the phylogenetic tree were removed) revealed that *SSL* gene family contained two highly divergent clusters, Cluster I and Cluster II, and multiple subfamilies. Cluster I comprised 2 subfamilies with each containing homologs from the major clades of land plants, including eudicots and monocots; Cluster II contained 5 subfamilies, among which sub-family VII (Sub-VII) was only composed of the homologs from eudicots but the other 4 subfamilies included homologs from both eudicots and monocots. Transcriptome analysis revealed that both parasitic plants contain multiple *SSL* copies, 7 copies in *O. aegyptiaca* belonging to 6 subfamilies and 5 copies in *C. australis* from 5 subfamilies.

Sub-VII contained 2 clades, Clade I and Clade II. Although Clade I contained homologs from diverse species of eudicots, Clade II only included *SSL* sequences from Brassicales and the 2 parasitic plants (Figure 1), and the *OaSSL* and *CaSSL* clustered with 3 Arabidopsis *SSL* genes with 100% bootstrap support. Thus, Clade II may be originated in Brassicales by gene duplication from Clade I before the divergence of Brassicales (the *SSL* genes in Clade I are hence called the original copies of the *SSL* genes in Clade II), and *OaSSL* and *CaSSL* were very likely derived from Brassicaceae by HGT.

**Figure 2:**
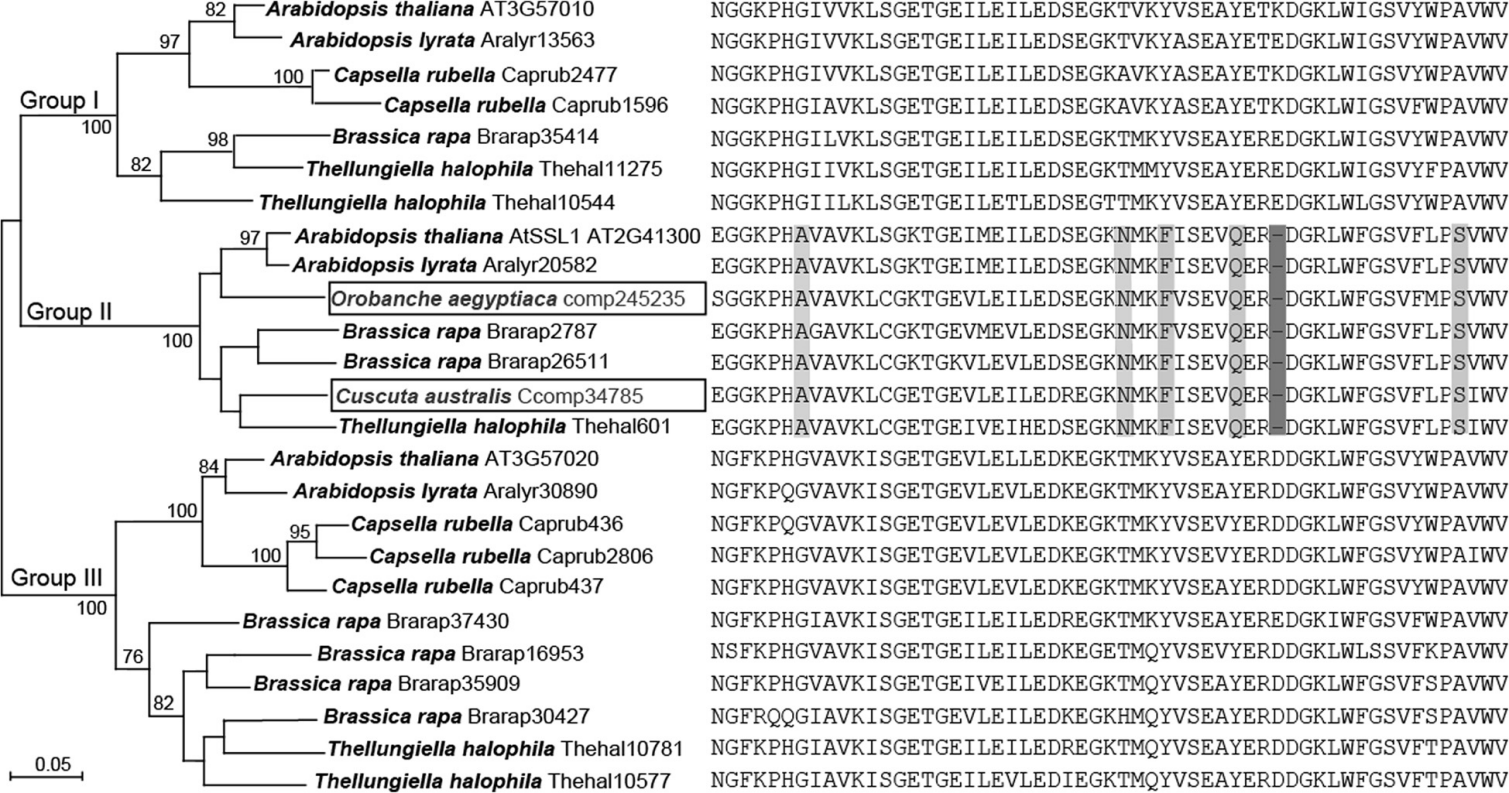
**Molecular phylogeny and partial alignment of the Brassicaceae-specific SSL proteins and the foreign SSLs in *O. aegyptiaca *and *C. australis*.** Sequence IDs are shown after the species names. Numbers above or below the branches indicate bootstrap support values of maximum likelihood no less than 75%. The sequences from parasitic plants *O. aegyptiaca* and *C. australis* are highlighted in boxes. The respective partial protein sequences are shown on the right side of the species names and sequence IDs. The amino acids and the single amino acid deletion uniquely conserved in Group II are shaded.

To gain more insight into the potential gene donors of the *SSL* genes in *Orobanche* and *Cuscuta*, we collected all *SSL* sequences from species with available genome sequences and also from the transcriptome assemblies of 3 shoot parasitic plants, *Cuscuta pentagona* (Convolvulaceae), *Cassytha filiformis* (Lauraceae), and *Pilostyles thurberi* (Apodanthaceae) from the 1KP Project (http://www.onekp.com/project.html), and 2 root parasitic plants, *Triphysaria versicolor* and *Striga hermonthica* from the Parasitic Plant Genome Project [40]. Phylogenetic analysis showed that only certain Brassicaceae *SSL* genes clustered closely with *OaSSL* and *CaSSL*. All these sequences clustered were used to infer the specific origins of foreign SSLs. The tree showed 3 groups, Group I to III, and the *SSL* genes from the two parasitic plants were assigned to Group II with 100% bootstrap support (Figure 2). Importantly, both *OaSSL* and *CaSSL* shared a single amino acid deletion and multiple unique amino acids that are uniquely conserved in Group II SSL proteins (Figure 2). These results indicated that *OaSSL* and *CaSSL* were originated from Brassicaceae, although we were not able to determine the exact donor species, since *OaSSL* and *CaSSL* showed ambiguous relationships with other species in Group II (Figure 2).

### The structures of Brassicaceae-specific *SSL* genes*,* OaSSL in O. *aegyptiaca,* and *CaSSL* in *C. australis*

Comparison of 23 *SSL* mRNA sequences with their respective genomic DNA sequences in 5 Brassicaceae species indicated that Brassicaceae *SSL* genes contained maximally 3 introns and among them 18 genes lacked the first intron. The positions of intron 2 and intron 3 were well conserved but the intron lengths varied within and between species (Additional file 2).

**Figure 3:**
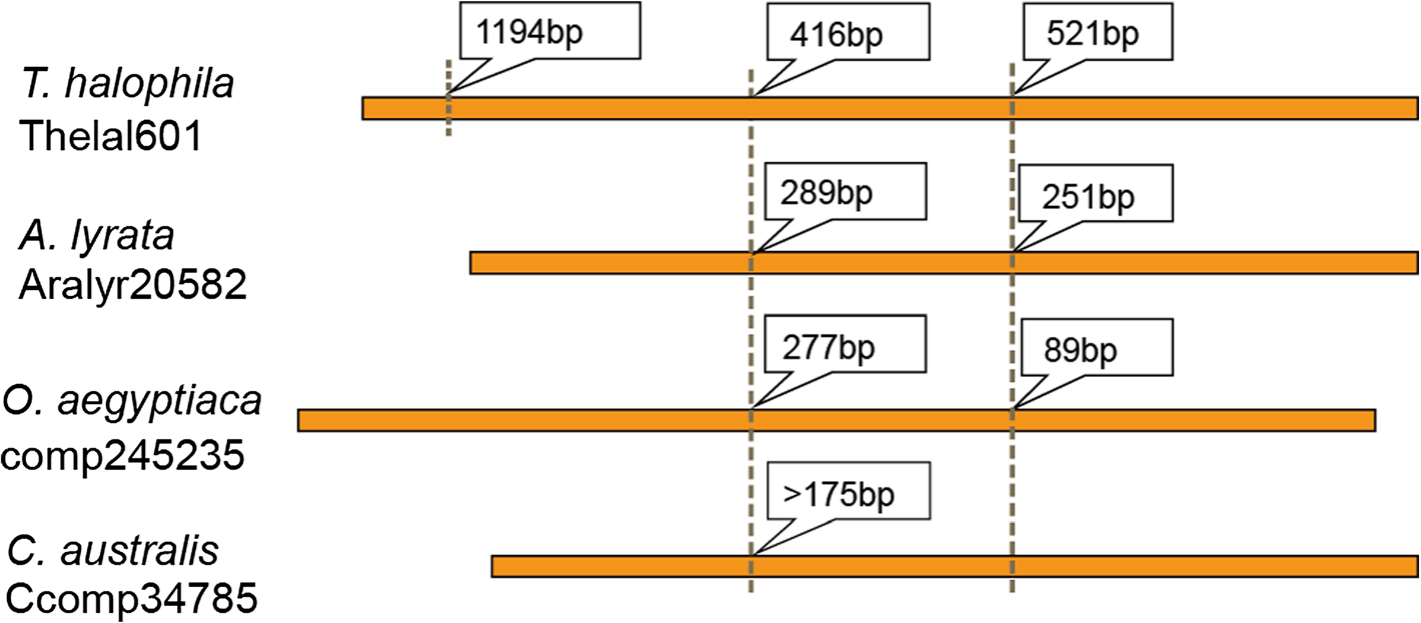
**Gene structures of representative Brassicaceae-specific *SSL*s and the foreign *SSL*s in *O. aegyptiaca *and *C. australis*.** The orange bars indicate the exons. The vertical dotted lines show the intron positions, with intron lengths marked in boxes. The intron length and position in *C. australis* were inferred from the difference in the two *CaSSL* mRNA isoforms.

Only one copy of *OaSSL* transcript was identified in the transcriptome of *O. aegyptiaca*. Using genomic DNA as the template, we cloned its genomic sequence (NCBI accession number: KF817597): it contained 2 introns, whose positions were identical to the intron 2 and 3 of the *SSL* genes in Brassicaceae (Figure 3). Analysis of *C. australis* transcriptome assembly revealed 2 isoforms of *CaSSL*, which were named Seq1 (NCBI accession number: KF817596) and Seq2 (NCBI accession number: KF817595). Seq1 shared 100% identity with Seq2 except that Seq1 had a 21-bp insertion (Additional file 3). The insertion was located at the same position as intron 2 in Brassicaceae-specific SSL genes, thus Seq1 was very likely originated from partial retention of intron 2. The putative protein sequences of Seq1 and Seq2 aligned well with SSLs from Brassicaceae without any frame shifts, suggesting that Seq1 and Seq2 encode functional proteins (Additional file 3). Genomic PCR was used to amplify the sequence of *CaSSL* gene, but various primers spanning the intron region failed to produce any products. We speculate that the intron in *CaSSL* may have complex secondary structures or may be very long. Intron 3 was obviously absent in *CaSSL* (NCBI accession number: KF817598, Figure 3).

By HGT, DNA can be transferred into nuclear, plastidal, or mitochondrial genomes of plants. All currently known Brassicaceae-specific *SSL* genes are encoded by the nuclear genomes. Nuclear or mitochondrial genomes from *Orobanche* or *Cuscuta* genera are not available, but a BLAST search of multiple chloroplast genomes of *Cuscuta* species [43] (chloroplast genome of *C. australis* is yet unavailable) indicated that none of the plastidal genomes contained homologs of *CaSSL* gene. We speculate that the *CaSSL* gene is likely in the nuclear genome of *C. australis*, although the possibilities of being in chloroplast or mitochondrial genome could not be completely ruled out. This might be also true for the *OaSSL* in *O. aegyptiaca*, since the current plastid genomes from *Orobanche* do not possess the *SSL* genes [44].

### Expression analysis of *AtSSL1* in *A. thaliana, OaSSL* in *O. aegyptiaca,* and *CaSSL* in *C. australis*

Because no functional studies have been reported for the Brassicaceae-specific *SSL* genes, we chose the *AtSSL1* gene in Arabidopsis (AT2G41300, in Clade II, Figure 1), which showed the highest similarities to *OaSSL* in *O. aegyptiaca* and *CaSSL* in *C. australis*, as a representative and investigated its expression profile in various developmental stages, and after diverse stress stimuli and treatments using GENEVESTIGATOR [45]. *AtSSL1* was expressed in all developmental stages at rather similar levels (Additional file 4). Strong up-regulation of *AtSSL1* expression was observed in inflorescence stem upon addition of 1-naphthaleneacetic acid and in roots inoculated with the nematode *Heterodera schachtii*; a large down-regulation was found in root after low oxygen treatment (Additional file 5). Therefore, we speculated that AtSSL1 might be involved in plant development and stress responses.

**Figure 4:**
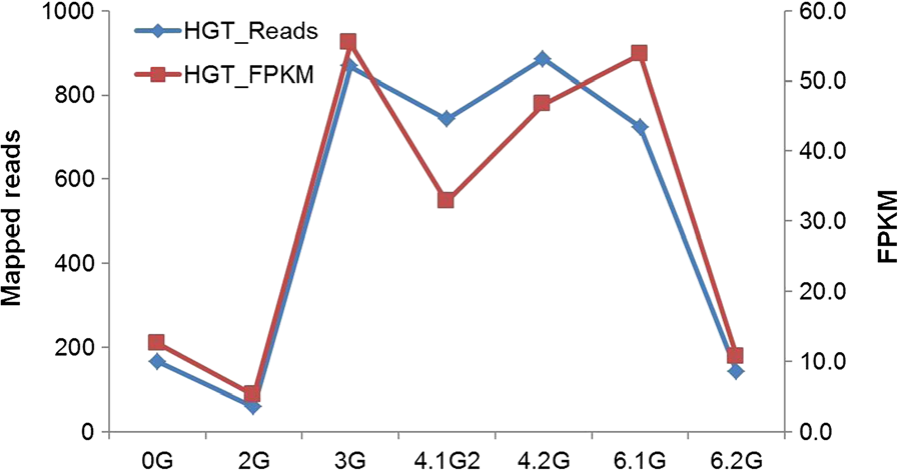
**Expression levels of the *OaSSL *gene at different developmental stages.** The numbers of mapped reads mapped to the cDNA were shown in the left Y-axis. The normalized expression levels were estimated by the fragments per kilo base of exon per million fragments mapped (FPKM, one fragment refers to a pair of reads from the paired-end data) in Illumina RNA-seq datasets that are from different developmental stages (PPGP). 0G, seeds imbibed, pre-germination; 2G, seedling after exposure to haustorial induction factors; 3G, haustoria attached to host root; early penetration stages, pre-vascular connection; 4.1G, early established parasite; parasite vegetative growth after vascular connection; 4.2G, spider stage; 5.1G, pre-emergence from soil - shoots; 5.2G, pre-emergence from soil - roots; 6.1G, post emergence from soil - vegetative structures, leaves/stems; 6.2G, post emergence from soil - reproductive structures, floral buds.

To gain insight into the potential function of *OaSSL* gene in *O. aegyptiaca*, we examined the expression profiles of *OaSSL* using the paired-end RNA-seq datasets in PPGP generated from different developmental stages of *O. aegyptiaca*[40] (Figure 4). *OaSSL* shows 3- to 5-fold increased transcript abundance during haustorial formation and vegetable growth stages (Figure 4), suggesting that *OaSSL* gene might perform functions in haustorial formation and vegetable growth.

**Figure 5:**
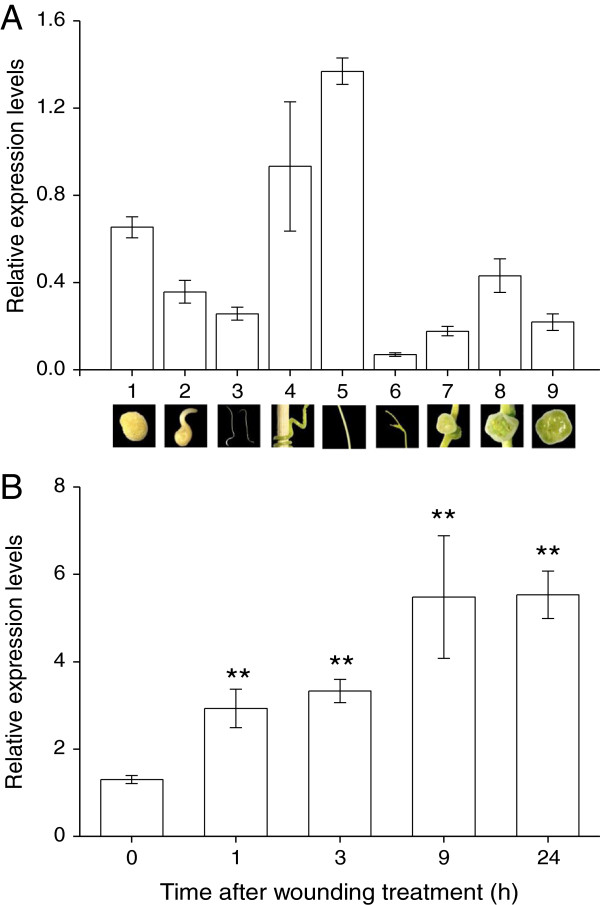
**Expression level analyses of the *CaSSL *gene in *C. australis *during different developmental stages and after wounding treatment by qRT-PCR. A**. Expression levels (±SE, n = 5) of the *CaSSL* gene at different developmental stages and in different organs. 1, seeds; 2, just germinated seeds; 3, seedlings; 4, pre-haustoria; 5, stems; 6, shoot tips; 7, floral buds; 8, flowers; 9, capsules. A picture of each developmental stage was shown below its expression labeling. **B**. Expression levels (±SE, n = 5) of the *CaSSL* gene after wounding treatment in stems. Asterisks indicate significant differences between treatment and 0-h time point (**P < 0.01; Student’s t-test).

Given that there are no public transcriptome databases available for *Cuscuta* spp., quantitative real time-PCR (qPCR) was done to measure the expression levels of *CaSSL* in *C. australis* in different developmental stages and after wound treatment. Because selection of suitable reference genes is important for qPCR assay, four commonly used reference genes, *actin*, *EF-1α*, *EF-TU*, and *PP2A*, were evaluated with geNorm [46], DeltaCT [47], BestKeeper [48], Normfinder [49], and RefFinder [50]. The result showed that *EF-1α* was stably expressed under all conditions (Additional file 6). Thus, *EF-1α* was used as the reference gene. *CaSSL* was expressed at all 9 different developmental stages or organs (Figure 5A), and the highest expression level was found in mature stems and the lowest was in shoot tips (the difference was 19.7-fold) (Figure 5A). Additionally the transcriptional levels of *CaSSL* before and 1, 3, 9, and 24 h after wounding were determined. The expression level of the *CaSSL* gene was up-regulated 1.3-fold 1 h after treatment and increased over time (increased 3.2-fold 24 h after treatment) (Figure 5B). These results imply that *CaSSL* might be involved in development and abiotic stresses, such as wounding.

### Functional divergence and selection analysis

Compared with its original gene copies (Clade I, Figure 1), the Brassicaceae-specific genes (Clade II, Figure 1) present as a relatively long branch in the phylogenetic tree, which suggests that functional divergence between the two subfamilies probably had occurred after the gene duplication event. To evaluate this scenario, two types of functional divergence (type I and type II) between these two subfamilies were estimated by posterior analysis with DIVERGE 2 [51]. Type I functional divergence (altered functional constrains) represents the conserved amino acid sites in one clade but highly variable in the other; type II functional divergence detected the conserved amino acid sites in both clades but with changed biochemical properties [52]. The coefficient of type I functional divergence was significantly greater than 0 (*θ*_1_=0.21040 ± 0.0343, LRT = 37.5692, p < 0.01) and 7 amino acid sites possessed a posterior probability higher than 0.7, which suggested that functional constrains on these 7 sites had shifted for most members between the two clades. No type II-related radical changes happened between the two clades after gene duplication. These results suggested that type I functional divergence was the dominant patter for the genes in Clade I and Clade II and altered site-specific selective constrains probable had led to group-specific functional changes between them.

We further investigated whether strong selection was involved in the evolution of the *SSL* genes in Clade I and II (Figures 1 and 2) using likelihood ratio tests developed by Yang et al. [53]. We firstly used the site models to detect whether positive selection occurred in some sites among the Brassicaceae-specific *SSL* genes, their original copies, and the foreign *SSL* genes in the two parasitic plants. No positive selection was detected in these genes (Additional file 7). Notably, the maximum likelihood estimates of the value under M0 model approached 0, 0.1777 in the large dataset (the Brassicaceae-specific genes and their original copies) and 0.1903 in the small dataset (the Brassicaceae-specific gene copies and two foreign *SSL* genes in the parasitic plants). The log-likelihood differences between models M0 and M3 were statically different for both the large dataset (LRT = 630.293, *p* < 0.01) and the small dataset (LRT = 387.605, *p* < 0.01) (Additional file 7). These suggested that relaxed purifying selection was the main driving force in the evolution of these genes.

Under the branch-site models, the null hypothesis and alternative hypothesis were examined by setting the Brassicaceae-specific *SSL* genes and each of the two parasitic genes separately as foreground. No evidence indicated that positive selection acted on the Brassicaceae-specific *SSL* genes and the *CaSSL* gene in *C. australis* (Additional file 8). However, two codon sites in the *OaSSL* gene of *O. aegyptiaca* have probably undergone positive selection (LRT = 5.754, *p* < 0.05) with more than 75% posterior probability (Additional file 8). Codon 181 encoded a glycine (G) or arginine (R) in the Brassicaceae-specific *SSL* genes, whereas it encoded an asparagine (N) in the *OaSSL* gene of *O. aegyptiaca*; site 267 was a serine (S) in the *OaSSL* gene, which showed a 0.975 posterior probability of positive selection, while it encoded an asparagine (N) or glutamic acid (E) in the Brassicaceae-specific genes (Additional file 9).

## Discussion

### The Brassicaceae-specific *SSL* genes and the alien origins of *SSL* genes in the parasitic plants *O. aegyptiaca* and *C. australis*

The *SSL* genes in plants constitute a large gene family that shows high levels of divergence among different subfamilies. Among them, Sub-VII is relatively special since it includes one clade (Clade I) containing *SSL* genes from different species of eudicots and the other clade (Clade II) only has species from Brassicales and the two parasitic plants studied in present work. It is very likely that *SSL* genes in Clade II were formed only in Brassicales via gene duplication of an ancestral *SSL* gene in Clade I.

Intriguingly, in this work we found that two *SSL* genes of Brassicaceae have been introduced to the two parasitic plants by horizontal gene transfer. This is strongly supported by the following evidence: 1) high identity values in the multiple sequence alignment, 2) shared indels and amino acids, and 3) robust bootstrap values in the phylogeny analyses. From these lines of evidence, the possibility of convergent evolution can be ruled out. Because *O. aegyptiaca* and *C. australis* belong to different taxa, the gene transfer events leading to the two foreign genes in the two distantly related parasitic plants should occur independently.

### The possible transfer mechanism

The existence of introns in *OaSSL* and *CaSSL* gene sequences indicates that both gene transfer events occurred not at the mRNA level but at the DNA level. Similarly, it was reported that the foreign albumin gene in *O. aegyptiaca* also contains introns and thus should be transferred as DNA [35]. Parasitic plants are known to transport thousands of mRNAs from their hosts [54,55]; however, it is likely that DNA, but not mRNA, transferred from a plant to another is the major form of HGT in plants. Probably this is because mRNA has to be reversed transcribed to DNA before they could be integrated into a plant genome and the activity of this type of reverse transcription is extremely low.

The occurrence of any HGT event at DNA level in plants requires multiple steps: 1) entering the cytoplasm after crossing the cell wall and cell membrane, 2) entering the nucleus through the nuclear membrane, 3) integration into the genome, 4) fixation and transfer to the next generation, 5) spread inside the population. The proposed mechanisms of HGT in plants include illegitimate pollination, vector-mediated, and plant-plant contact hypothesis [5,19]. Illegitimate pollination hypothesis presumes that the pollen grains of the gene donors can germinate on the stigma of the reproductive isolated species, after pollination, recombination may allow some foreign genes to be integrated into the other set of chromosomes, and hybrids with the same species and natural selection may lead to the retain of the target chromosome set with some foreign genes within the population. This HGT mechanism is considered to occur between closely related species, such as the HGT cases between *Setaria* and *Oryza*[25], between *Poa* and *Festuca*[56], or between C4 and C3 species in *Alloteropsis*[26]. Because both *O. aegyptiaca* and *C. australis* are distantly related with Brassicaceae, the illegitimate pollination hypothesis is very unlikely.

The vector-mediated transfer hypothesis assumes that viruses, pathogens, endophytes, or other media act as vectors to transfer the foreign DNA into the recipients. Because most vectors have limited host ranges, vector-mediated HGT from Brassicaceae (or from Fabaceae, in the case of *albumin 1*[35]) to Orobanchaceae and Convolvulaceae appears to be unlikely. Only vectors with a wide host range, such as apple latent spherical virus [57], may be able to mediate these transfer events. However, no plant viruses carrying the foreign genes have been discovered so far. This is possibly due to fast replication of virus which may cause loss of foreign genes during amplification. Evidence of virus carrying foreign genes is needed to confirm the vector-mediated HGT as a plausible mechanism.

Plant-Plant contact hypothesis suggests that the foreign DNA is imported into the recipients via the direct contact between plants, such as the long-term intimate contact in parasitic systems. Parasitism establishes a functional symplastic pathway between parasitic plant and host, allowing transport of nutrients, RNAs [54,58], proteins [59-61], viruses [62], and phytoplasma [63] from the hosts to the parasites. Currently, no direct evidence indicates that host DNA molecules can be transported into parasitic plants, but movement of large DNA fragments and even whole chloroplasts has been observed in the graft regions between the same species [64] or between sexually incompatible species [65]. The connections between parasitic plants and their hosts are highly similar to the artificial graft junctions; thus, regardless of whether the transfer of DNA is limited to cell-to-cell in a short range or can occur in relatively long distances, the fusion of host and parasite vascular systems provides possibilities for foreign DNA to travel to the recipients. Because the concentration of imported macromolecules also decreases along the stems [55,61], the probability for the integration of foreign genetic materials decreases with the distance from the haustoria. We hypothesize that Brassicaceae *SSL* genes were firstly integrated to the cells of *O. aegyptiaca* and *C. australis* haustoria or nearby tissues, and certain cells somehow developed into floral meristems, allowing the host genes to pass to the next generations.

### Functional implication of the foreign *SSL* genes in *O. aegyptiaca* and *C. australis*

The initial biochemical evidence for the function of a *SSL* gene was from *Rauvolfia Serpentina*, in which the activity of a strictosidine synthase (STR) that catalyzes the stereospecific condensation of tryptamine and secologanin to form strictosidine (Pictet-Spengler reaction) was detected [66], and later, the coding gene was cloned [67]. Because strictosidine is the key intermediate in indole alkaloid biosynthesis, which is the precursor of several clinically useful anti-cancer alkaloids, the STR genes were successively elucidated in *Catharanthus roseus*[68], *Rauwolfia verticillata*[69], and *Ophiorrhiza japonica*[70]. However, compared with the catalytic feature and the active site information in the 3D-structure of STRs [71,72], Hickes et al. suggested that the great majority of sequences annotated as STR do not catalyze the Pictet-Spengler reaction [73].

We added all sequences encoding proteins with confirmed STR activity in our comprehensive phylogenetic analysis, and they clustered as a highly supported clade (Figure 1). One sequence from *Vitis vinifera* (CAN77945) is related to those with verified STR activity in our phylogenetic tree. No STR activities but hydrolytic activities were detected in this *SSL* gene of *V. vinifera*[73]. Given the low sequence similarities and distant relationships of the genes in Clade II to the real STRs, the Brassicaceae-specific and the foreign *SSL* genes in the parasitic plants probably do not catalyze the Pictet-Spengler reaction. We found that the *SSL* genes in Arabidopsis and the parasitic plants are actively transcribed in multiple developmental stages and following different treatments, but their biological functions need to be further studied.

Parasitic plants face at least two challenges. First, they need to conquer host defenses while at the same time absorbing nutrients from the hosts; second, they have to resist herbivores, bacteria, fungi, or abiotic environment stresses, such as wounding. Previous work had indicated that transferred genes in nematodes from microorganism may have enabled the parasites to modulate the host defense systems [74]. Parasitic oomycetes probably acquired certain secretory proteins from fungi, which inhabit the same niche with the oomycetes, and thus are able to suppress certain host defense responses [75]. Data-mining in the public microarray database revealed that the most related Arabidopsis *AtSSL1* also responds to the treatments of hormones, abiotic and biotic stresses. *OaSSL* in *O. aegyptiaca* showed different expression levels at different developmental stages and had undergone positive selection. Similarly, in *C. australis*, *CaSSL* also exhibited developmental stage- and organ-specific expression levels; furthermore, wounding transcriptionally activated *CaSSL*. Therefore, the foreign *SSL* genes in the two parasitic plants might be involved in plant development and secondary metabolism, which is normally associated with herbivore and pathogen resistance [76,77] or other environmental stresses [78].

## Conclusion

HGT from the host nuclear genome to parasitic plants has been rarely reported so far, here we provide solid evidence indicating that two nuclear-encoded *SSL* genes from Brassicaceae species were co-opted by two distantly related holoparasitic plants, the root parasitic plant *O. aegyptiaca* and the shoot parasitic plants *C. australis*. Thus, the physical connection with host plant and the parasitic lifestyle including the transport of host nutrients and macromolecules may give parasites the opportunity to obtain host genes by HGT, and HGT between host and parasitic plant nuclear genomes may not be very rare. Our analysis also suggests a potential role of the horizontally transferred genes in the evolution and adaptation to parasitic lifestyle or environment.

## Methods

### Data sources

All the 12 Illumina datasets from *O. aegyptiaca* and the original transcriptome assembly OrAeBC4 were retrieved from the Root Parasitic Plant Genome Project (PPGP) website (http://ppgp.huck.psu.edu/). The transcriptome assemblies of other parasitic plants from PPGP, *Striga hermonthica* (StHeBC2), *Triphysaria versicolor* (TrPuRnBC1), and *Triphysaria pusilla* (TrVeBC2), were also downloaded. The protein database needed in AlienG [41] for BLAST search included NCBI non-redundant (nr) database (Sep. 2012) and all predicted proteins of 22 plant genomes which were available at Phytozome (version 9.0) [39] but absent from nr database. The nucleotide database included NCBI nucleotide collection (nt) and all the predicted transcripts from 22 plant genomes from Phytozome. The databases in the 1 KP project (http://www.onekp.com/project.html; which includes the transcriptome assemblies from *Cuscuta pentagona*, *Cassytha filiformis*, and *Pilostyles thurberi*), PlantGDB (http://www.plantgdb.org), and SOL Genomics Network (http://solgenomics.net) were searched online.

### Transcriptome screening of horizontally transferred genes in *O. aegyptiaca*

Because parasitic plants transport thousands of mRNAs from their hosts, the dataset used for the original transcriptome assembly includes samples attached to the hosts, and therefore the assembly contains contamination from the host mRNAs. To remove the contaminated sequences, the reads from two samples that have not attached to the hosts, seed germination (OrAe0G) and germinated seed, radicle emerged, and pre-haustorial growth (OrAe1G), were mapped to the unique sequences using RSEM [79] and the assembled sequences with mapped reads were kept. As for the assembled sequences with length no less than 300 nt, the potential ORFs were predicted locally by OrfPredictor [80] using default settings, and the amino acid sequences whose lengths were not less than 100 were screened for genes with potential alien origins using AlienG [41]. The alien origin of a gene was predicted if the score ratio of the first non-Lamiales hit to the first Lamiales hit was more than 1.2. The obtained candidates were further filtered to exclude those with *M. guttatus* affiliation in their corresponding cDNA sequences by BLAST search, since some non-coding genes were found to be rigidly translated into proteins.

### Total RNA extraction, cDNA library construction and transcriptome sequencing of *C. australis*

Seeds of *C. australis* were treated with sulfuric acid for 20 min to loosen the seed coat, and then the sulfuric acid was removed by extensive rinsing with water. The seeds were kept at 25°C for 3–4 days on wet filter paper until the seedlings were ~ 4 cm long. The seedlings were gently fastened to young soybean (*Glycine max*) plants (~ 20–25 cm tall; cultured at 26 ± 2°C,12 h light) using cotton threads and water was sprayed to these dodder seedlings 2–3 times/day to prevent them from drying out until parasitization was established. Total RNA was extracted from *C. australis* stem using the RNAeasy Plant Mini Kit (Qiagen) following the manufacturer’s instructions. Potential DNA contamination was removed by DNase treatment (Qiagen). The cDNA library was constructed using the Truseq RNA and DNA Sample Prep Kit following the user manual except that the insert fragments after adaptor ligation were set to 300 bp (the cDNA insertion length is about 180 bp). The paired short reads (2 × 101 bp) were generated on an Illumina Hiseq™ 2Clade I instrument. Image deconvolution and quality calculation were conducted by using the Illumina GA pipeline 1.6.

### Identification of *SSLs* in the transcriptomes of the two parasitic plants

The potential adaptor tags and their following sequences were removed from all raw reads in the Illumina datasets from *O. aegyptiaca* and *C. australis* using cutadapt [81]. The clean reads were obtained by further filtering out bases with low quality values (below 25) using Btrim [82]. *De novo* assembly of RNA-seq data was carried out using the Trinity software package [42] with the maximum length expected between fragment pairs set to 300. The foreign *SSL* cDNA fragment from the assembly of *O. aegyptiaca* in PPGP was used to search against the home made Trinity assembly of this species. The obtained putative full-length cDNA was used to search against the transcriptome assemblies of *C. australis* and other parasitic plants, including *Striga hermonthica*, *Triphysaria versicolor*, *Triphysaria pusilla*, *Cuscuta pentagona*, *Cassytha filiformis*, and *Pilostyles thurberi*.

### Expression level estimation of *OaSSL* gene by transcriptome analysis, visualization of mapping of RNA-seq Reads to the assembled sequences, and microarray expression analysis of *Arabidopsis AtSSL1* gene

The expression levels of the foreign *SSL* gene in *O. aegyptiaca* in different tissues and developmental stages were estimated using RSEM [79] by mapping all the clean reads obtained above to our Trinity assemblies in *O. aegyptiaca*. To check the possible assembly errors, mapping of all the clean reads from all these datasets to the foreign *SSL* cDNAs was visualized with Tablet [83]. Web-based expression analysis of *A. thaliana AtSSL1* gene (AT2G41300) in different tissues and multiple development stages and under various treatments was performed using GENEVESTIGATOR (https://www.genevestigator.com/gv/, [45].

### Cloning of *SSL* genes in parasitic plants

To obtain the genomic sequences of the foreign *SSL* genes, genomic DNA was extracted from 3 mg dried seeds of *O. aegyptiaca* and 0.1 g stem of *C. australis* using a modified cetyltrimethylammonium bromide (CTAB) method [84]. Specific primer pairs oaeF2/R1370 (5′-GCCACGTAGTGTGAAGCTT-3′/5′-CATCTCTTTCTTGAACCTCAC-3′) and cusF52/ R1470 (5′-AGGAACGAAGGGAGTATTT-3′/5′-TATTCATGAACTTCCGATATGG-3′) were designed according to the *SSL* cDNA sequences in *O. aegyptiaca* and *C. australis*, respectively. Amplification products of expected sizes were extracted using AxyPrep DNA Gel Extraction Kit (Axygen Bioscienes), cloned into the pMD18-T vector (Takara), and sequenced.

### qRT-PCR of the *CaSSL* gene in *C. australis*

Fresh tissues were collected from 9 different development stages: dry seeds, germinated seeds with 2 mm hypocotyl and 3-cm hypocotyl, pre-haustoria (2 d after attaching to pseudo-hosts, bamboo sticks), and mature stems (at least 4 cm far from shoot tips), shoot tips, floral buds, flowers and capsules. For wounding treatment, 2 cm long dodder stems that were 4 cm far from shoot tips were wounded by four rows of thin wires (diameter 0.1 mm) from an iron wire gauze. Samples were harvested 0, 1, 3, 9, and 24 h after treatment. Five biological replicates were taken for each time point. Total RNA from each sample was extracted with Fruit-mate™ and RNAiso Plus (TaKaRa) following the manufacturer’s instructions. RNA concentrations were quantified and 500 ng of each RNA sample was reverse-transcribed using oligo_(dT)_18_ and RevertAid™ H Minus Reverse Transcriptase (Fermentas) in a total volume of 10 μL. The obtained cDNA samples were diluted to 25 μL. Specific primer pair RT-cus34-F2/RT-cus34-R1 (5′-CTGCGACGGTTACCTTGGAA-3′/5′-CCACCATGTCCACCACTTTCT-3′) was designed according to a shared region by the 2 mRNA isoforms. q-PCR was used to characterize the expression of the foreign *SSL* gene in *C. australis* from different development stages and wounding treated stems on an CFX Connect™ Real-Time System (BIO-RAD) using iTaq™ Universal SYBR Green Supermix (BIO-RAD) following the manufacturer’s instructions. The reference gene for normalizing cDNA concentration variations was selected from four candidates, *actin*, *elongation factor 1α* (*EF-1α*), *elongation factor Tu* (*EF-Tu*), and *serine/threonine-protein phosphatase 2A* (*PP2A*). Their Ct values in all the samplings were recorded using the same amount of total RNA. The most stably expressed gene was selected by comparing their Ct values of samples from different developmental stages and wounding treatment using the online server RefFinder (http://www.leonxie.com/referencegene.php). Changes in expression levels of the foreign *SSL* gene were assessed using the comparative CT method. Differences in expression levels of SSL induced by wounding treatment were determined by Student’s *t* test.

### Phylogenetic analyses

Protein sequences were sampled from representative plants in Phytozome by BLAST search using the *OaSSL* gene in *O. aegyptiaca* as the query. Because *OaSSL* homolog genes have multiple copy numbers in each species, we constructed a bootstrap NJ tree using ClustalX2 [85] and only kept a representative sequence for each species in each branch. The homologs in *O. aegyptiaca* and *C. australis* were obtained by tblastn search against the RNA-seq assembly constructed in-house with the horizontal transferred gene in *O. aegyptiaca* as the query.

All these collected protein sequences were aligned using ClustalX2 [86]. We visually inspected the alignments and performed manual refinement. Gaps and ambiguously sites were removed from the alignment. The most optimal model of protein substitution matrix and rate heterogeneity was determined by ModelGenerator (v_851) [87]. Phylogenetic trees were reconstructed with a maximum likelihood method using PHYML 3.0 [88]. Bootstrap analyses used 100 pseudo-replicates. The topology structures of trees were viewed and edited with NJplot [89].

### Analysis of functional divergence

The coefficients of type I and type II functional divergence between the Brassicales-specific genes and its original copies were estimated by DIVERGE 2.0 [51]. Type I functional divergence (*θ*_1_) alters functional constraints on some sites after gene duplication, and type II functional divergence (*θ*_11_) results in radical change in amino acid properties between the two duplicated copies. A likelihood ratio test was conducted using distribution with 1 degree of freedom (DF).

### Detection of positive selection

The corresponding cDNAs were obtained by BLAST search against all the transcripts from Phytozome and the transcriptome assemblies from the parasitic plants. The selection pressure acting on the *SSL* coding regions was analyzed by calculating the rate ratio of nonsynonymous to synonymous substitution (or) with the program *codeml* implemented in PAML v4.7 [53]. We created large and small two datasets. The large dataset included the Brassicales-specific gene copies and their original gene copies; the small dataset included the Brassicales-specific gene copies and the foreign *SSL* genes in the two parasitic plants. The online server PAL2NAL [90] was used to convert the protein sequence alignments into the corresponding codon alignments, which were used as the input files of *codeml* in PAML. Unrooted NJ trees with branch lengths were produced using ClustalX 2.1 [85] based on the protein alignments and were fed to *codeml* in PAML.

We analyzed the site models and branch-site models on the large and small datasets with likelihood ratio tests (LRT), respectively. As for the site models, the LRT of M0-M3 comparison was used to test variable among sites with DF set to 3. The LRTs of M1a-M2a and M7-M8 comparison were used to test positive selection with DF set to 2. As for the branch-site models, the null hypothesis was compared with the alternative hypothesis to test whether positive selection acted on the Brassicales-specific *SSLs* and the foreign *SSLs* in the two parasitic plants, which were used as foreground in their own analysis. We chose the Bayes Empirical Bayes (BEB) analysis in *codeml* to calculate the posterior probability of sites undergone positive selection in the interested lineages.

## Competing interests

The authors declare that they have no competing interests.

## Authors’ contributions

Conceived and designed the study: GS and JQW. Generated the data and performed the analysis: GS DZ JQ JY TS ZY JSW LW. Contributed reagents/materials/analysis tools: JLH SL JFW. Wrote the paper: GS JQW. All authors read and approved the final manuscript.

## Supplementary Material

Additional file 1 **Schematic outline of the identification of horizontally transferred genes in *O. aegyptiaca *** **transcriptomes.** Click here for file

Additional file 2 **Gene loci and the structures of the Brassicaceae-specific *SSL *** **genes.** Click here for file

Additional file 3 **The gene Structure and its two mRNA isoforms in *CaSSL***. Click here for file

Additional file 4 **The *AtSSL1 *** **expression levels of *A. thaliana *** **in different developmental stages calculated by GENEVESTIGATOR.** Click here for file

Additional file 5 **The *AtSSL1 *** **expression changes of *A. thaliana *** **upon different treatments calculated by GENEVESTIGATOR.** Click here for file

Additional file 6 Primer sequences of four candidate reference genes and their ranking orders of table expression evaluated by RefFinder. Click here for file

Additional file 7 Results of positive selection using site models. Click here for file

Additional file 8 Results of positive selection using branch-site models. Click here for file

Additional file 9 **Sites undergone positive selection in the *OaSSL *** **gene of *O. aegyptiaca.***
Click here for file
