# Supplementary material for: Root parasitic plant Orobanche aegyptiaca and shoot parasitic plant Cuscuta australis obtained Brassicaceae-specific strictosidine synthase-like genes by horizontal gene transfer
Source: BMC Plant Biol. 2014 Jan 13;14:19. doi: 10.1186/1471-2229-14-19 (PMC3893544; doi:10.1186/1471-2229-14-19)
Supplement: Additional file 1 — Schematic outline of the identification of horizontally transferred genes in O. aegyptiaca transcriptomes. [file 1471-2229-14-19-S1.pdf]

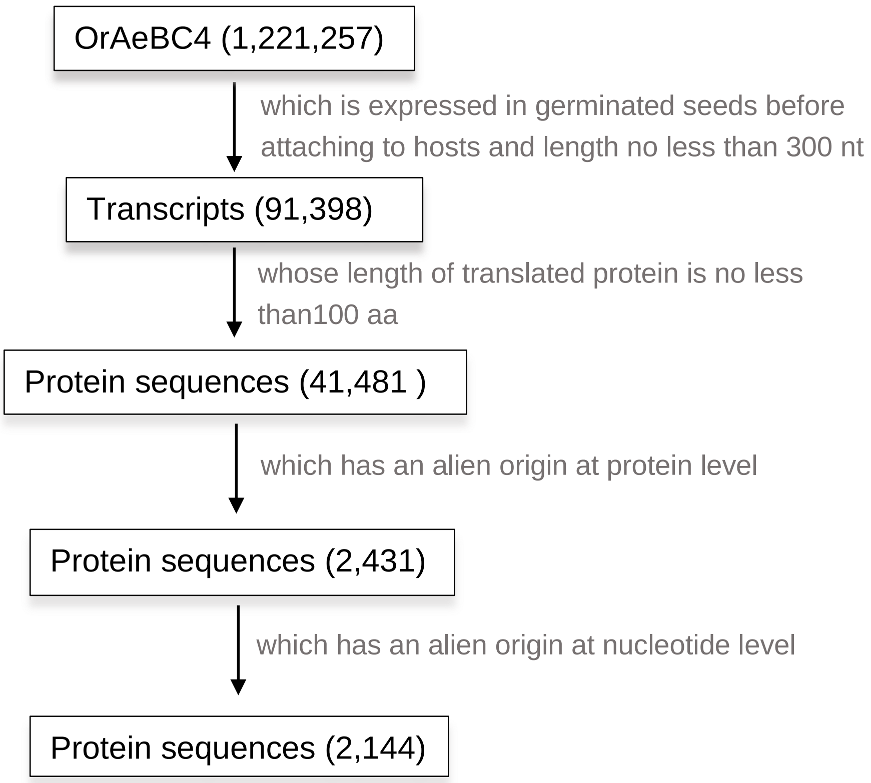

Additional File 1. Schematic outline of the identification of horizontally transferred genes in *O. aegyptiaca* transcriptomes.

The numbers of sequences passed each filtration are shown in parentheses.
