# Supplementary material for: Root parasitic plant Orobanche aegyptiaca and shoot parasitic plant Cuscuta australis obtained Brassicaceae-specific strictosidine synthase-like genes by horizontal gene transfer
Source: BMC Plant Biol. 2014 Jan 13;14:19. doi: 10.1186/1471-2229-14-19 (PMC3893544; doi:10.1186/1471-2229-14-19)
Supplement: Additional file 2 — Gene loci and the structures of the Brassicaceae-specific SSL genes. [file 1471-2229-14-19-S2.pdf]

Additional File 2. Gene loci and the structures of the Brassicaceae-specific *SSL* genes.

**Species**	**IDs**	**Phytozome_IDs**	**Coordinates**	**Length (bp)**						
				**Exon1**	**Exon2**	**Exon3**	**Exon4**	**Intron1**	**Intron2**	**Intron3**
A. lyrata	Aralyr13563	938669|PACid:16047309	scaffold_5:18193724-18192360		308	255	594		113	99
A. lyrata	Aralyr20582	321816|PACid:16054328	scaffold_4:20126225-20124576		284	255	574		291	250
A. lyrata	Aralyr30890	938670|PACid:16064636	scaffold_5:18198679-18196742	277	181	21	594	664	55	152
A. thaliana	Aratha5256	AT2G41300.1|PACid:19643202	Chr2:17216292-17214237	57	302	255	646	103	261	438
A. thaliana	Aratha23807	AT3G57010.1|PACid:19661753	Chr3:21097451-21095654		422	255	789		139	197
A. thaliana	Aratha26514	AT3G57020.1|PACid:19664460	Chr3:2100360-21098315		341	255	774		533	147
B. rapa	Brarap2787	Bra004614|PACid:22686296	A05:1065033-1062979		284	255	571		368	581
B. rapa	Brarap16953	Bra003263|PACid:22700462	A07:12261697-12259770		284	255	574		475	344
B. rapa	Brarap26511	Bra000223|PACid:22710020	A03:9990231-9988008		284	255	571		657	461
B. rapa	Brarap30427	Bra007293|PACid:22713936	A09:28429126-28427067		281	255	574		231	723
B. rapa	Brarap35414	Bra014655|PACid:22718923	A04:2043037-2044716		302	255	589		287	251
B. rapa	Brarap35909	Bra014652|PACid:22719418	A04:2030142-2033263		284	255	574		1707	306
B. rapa	Brarap37430	Bra034344|PACid:22720939	A04:12033036-12034765		284	255	574		485	136
C. rubella	Caprub436	Carubv10017468m|PACid:20886048	scaffold_5:11357994-11356407		402	255	599		175	161
C. rubella	Caprub437	Carubv10017465m|PACid:20886049	scaffold_5:11355209-11353024		558	255	583		626	168
C. rubella	Caprub1596	Carubv10017457m|PACid:20887215	scaffold_5:11349933-11348445		287	255	649		133	169
C. rubella	Caprub2477	Carubv10017448m|PACid:20888101	scaffold_5:11346881-11345313		308	255	669		147	194
C. rubella	Caprub2806	Carubv10017464m|PACid:20888433	scaffold_5:11363796-11362111		345	255	712		215	163
T. halophila	Thehal601	Thhalv10016720m|PACid:20180072	scaffold_10:11153980-11150593	122	309	255	571	1196	418	523
C. rubella	Thehal10544	Thhalv10006039m|PACid:20190080	scaffold_19:2356012-2357795		358	255	863		194	118
C. rubella	Thehal10577	Thhalv10006018m|PACid:20190113	scaffold_19:2349997-2352569	32	354	255	756	255	624	301
C. rubella	Thehal10781	Thhalv10006043m|PACid:20190317	scaffold_19:2353055-2355106		366	255	780		524	131
C. rubella	Thehal11275	Thhalv10005873m|PACid:20190812	scaffold_19:2344725-2347134	491	297	255	706	428	153	86
