# Supplementary material for: Root parasitic plant Orobanche aegyptiaca and shoot parasitic plant Cuscuta australis obtained Brassicaceae-specific strictosidine synthase-like genes by horizontal gene transfer
Source: BMC Plant Biol. 2014 Jan 13;14:19. doi: 10.1186/1471-2229-14-19 (PMC3893544; doi:10.1186/1471-2229-14-19)
Supplement: Additional file 3 — The gene Structure and its two mRNA isoforms in CaSSL . [file 1471-2229-14-19-S3.pdf]

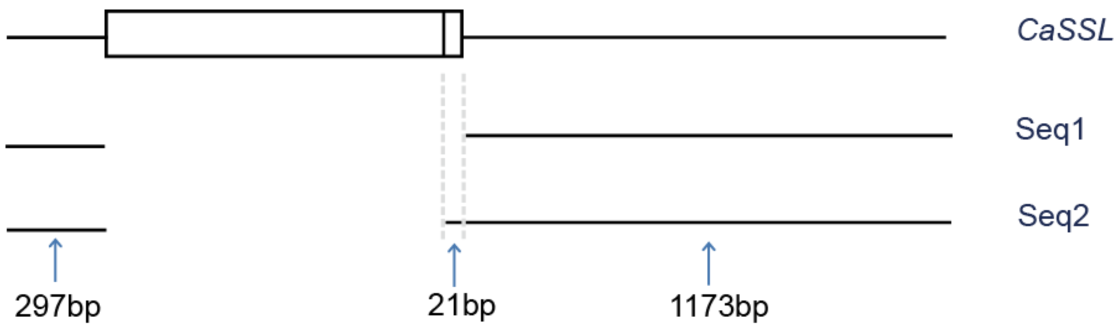

Additional File 3. The gene Structure and its two mRNA isoforms in *CaSSL*.

Boxes and horizontal lines indicate introns and exons, respectively. The short vertical line within the box indicates alternative splicing sites. The Dotted lines show the relative positions of alignment. The lengths of each fragment are indicated below. The intron length is unknown. The difference of Seq2 with Seq1 lies in a 21-bp indel.
