# Supplementary material for: Root parasitic plant Orobanche aegyptiaca and shoot parasitic plant Cuscuta australis obtained Brassicaceae-specific strictosidine synthase-like genes by horizontal gene transfer
Source: BMC Plant Biol. 2014 Jan 13;14:19. doi: 10.1186/1471-2229-14-19 (PMC3893544; doi:10.1186/1471-2229-14-19)
Supplement: Additional file 4 — The AtSSL1 expression levels of A. thaliana in different developmental stages calculated by GENEVESTIGATOR. [file 1471-2229-14-19-S4.pdf]

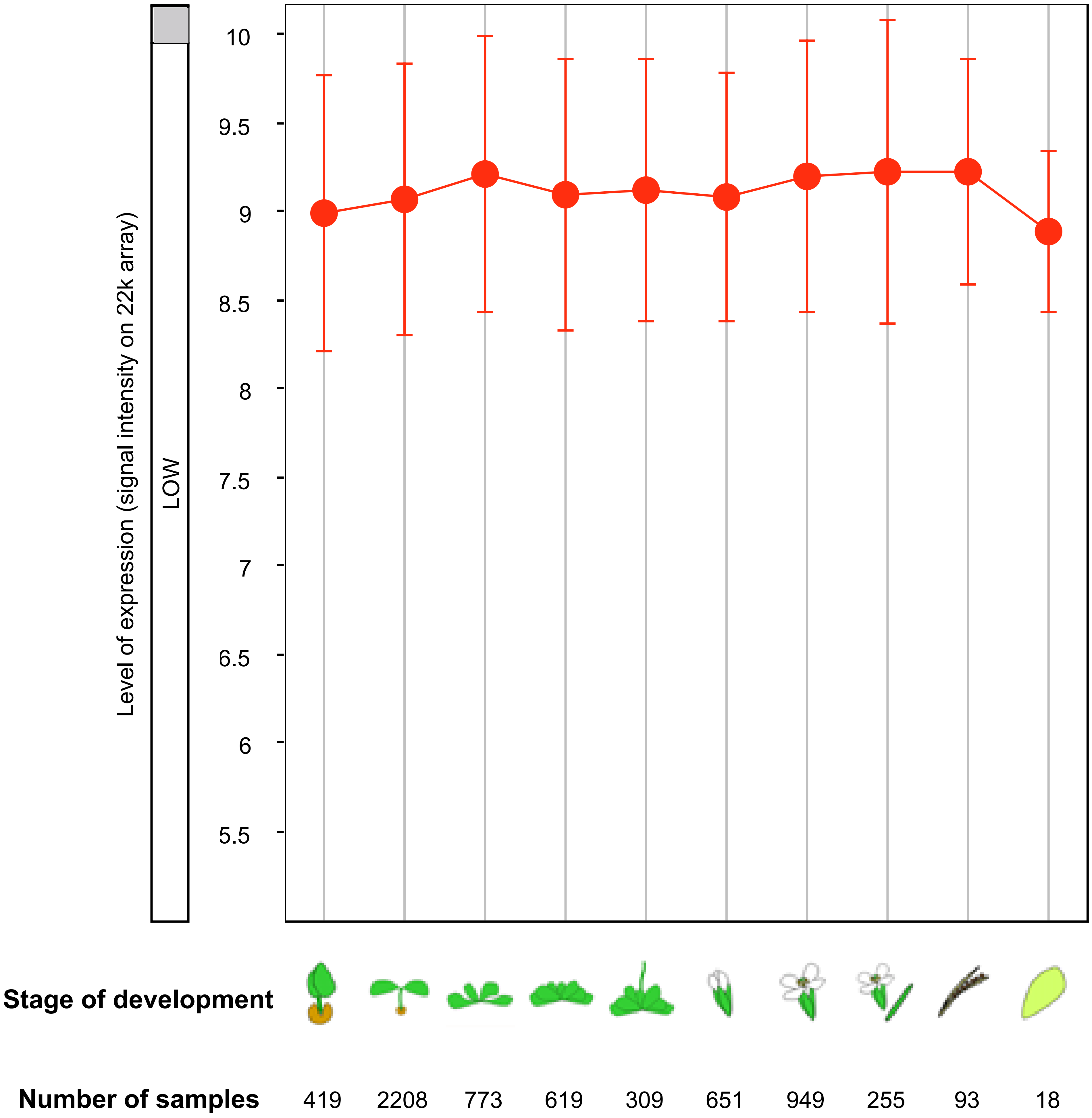

Level of expression (signal intensity on 22k array)
LOW
10
9.5
9
8.5
8
7.5
7
6.5
6
5.5
Stage of development
Number of samples
419
2208
773
619
309
651
949
255
93
18

Additional File 4. The *AtSSL1* expression levels of *A. thaliana* in different developmental stages calculated by GENEVESTIGATOR.

The expression level for each developmental stage was calculated from sampling pools of the stage. A picture and sampling pool number of each developmental stage were shown below its expression labeling.
