# Supplementary material for: Root parasitic plant Orobanche aegyptiaca and shoot parasitic plant Cuscuta australis obtained Brassicaceae-specific strictosidine synthase-like genes by horizontal gene transfer
Source: BMC Plant Biol. 2014 Jan 13;14:19. doi: 10.1186/1471-2229-14-19 (PMC3893544; doi:10.1186/1471-2229-14-19)
Supplement: Additional file 6 — Primer sequences of four candidate reference genes and their ranking orders of table expression evaluated by RefFinder. [file 1471-2229-14-19-S6.pdf]

Additional File 6. Primer sequences of four candidate reference genesand their ranking orders of table expression evaluated by RefFinder.

The ranking orders of the four candidate reference genes are given by four popular tools that evaluate reference gene expression, geNorm, DeltaCT, BestKeeper, and Normfinder. The recommended comprehensive ranking is obtained with RefFinder by assigning an appropriate weight to each individual gene (based on the rankings from the four tools) and calculating the geometric mean of their weights for the overall final ranking (indicated in brackets).

**Candidate reference genes**	**Forward primer sequence**	**Backward primer sequence**	**geNorm**	**Delta CT**	**Best Keeper**	**Norm finder**	**Recommended comprehensive ranking (RefFinder)**
EF-1α	TCAGACTGTTGCTGTGGGTG	CCCTTGTGCGGTTCACTTCT	1	1	1	1	1 (1.25)
EF-Tu	CAGAGCCTGAAACAATGGGAAT	CGAATTGGAGGTAAGGGAGC	2	2	2	2	2 (2.00)
actin	CTCAGCACCTTCCAGCAGA	AAGAGGCAAACTATTGTAGCATCT	3	4	3	4	4 (3.94)
PP2A	ATCCGCACTTCAGCCTCGTT	GAGGCAGTTGGACCTGAAG	4	3	4	3	3 (3.84)
