# Supplementary material for: Root parasitic plant Orobanche aegyptiaca and shoot parasitic plant Cuscuta australis obtained Brassicaceae-specific strictosidine synthase-like genes by horizontal gene transfer
Source: BMC Plant Biol. 2014 Jan 13;14:19. doi: 10.1186/1471-2229-14-19 (PMC3893544; doi:10.1186/1471-2229-14-19)
Supplement: Additional file 7 — Results of positive selection using site models. [file 1471-2229-14-19-S7.pdf]

Additional File 7. Results of positive selection using site models

Branches	Models	$\ln L$	Parameters	PSS[3]
Large[1]	M0	-13826.6585	$\omega = 0.1777$	-
	M3	-13511.5122	$p_0 = 0.2974, p_1 = 0.4798, p_2 = 0.2228$<br>$\omega_0 = 0.0151, \omega_1 = 0.1715, \omega_2 = 0.5014$	n.d.[4]
	M1a	-13681.8672	$p_0 = 0.8422, p_1$=0.1578<br>$\omega_0 = 0.1451, \omega_1 = 1.0000$	-
	M2a	-13681.8672	$p_0 = 0.8422, p_1 = 0.1578, p_2 = 0.00000$<br>$\omega_0 = 0.1451, \omega_1 = 1.0000, \omega_2 = 27.2532$	n.d.
	M7	-13509.8118	$\beta(0.6711, 2.6316)$	-
	M8	-13509.8124	$\beta(0.6711, 2.6317)$<br>$p_1 = 0.0000, \omega$=1.0000	n.d.
Small[2]	M0	-10086.6556	$\omega = 0.1903$	-
	M3	-9892.8530	$p_0 = 0.4314, p_1 = 0.3686, p_2 = 0.2000$<br>$\omega_0 = 0.0210, \omega_1 = 0.2345, \omega_2 = 0.5702$	n.d.
	M1a	-9981.9712	$p_0 = 0.7960, p_1$= 0.2040<br>$\omega_0 = 0.1255, \omega_1 = 1.0000$	-
	M2a	-9981.9712	$p_0 = 0.7960, p_1 = 0.2040, p_2 = 0.0000$<br>$\omega_0 = 0.1255, \omega_1 = 1.0000, \omega_2 = 36.8437$	n.d.
	M7	-9894.3065	$\beta(0.5060, 1.8960)$	-
	M8	-9894.3098	$\beta(0.5099, 1.8960)$<br>$p_1 = 0.0000, \omega$=3.8696	n.d.

[1], The sequences used in the large data include the Brassicaceae-specific gene copies (sequences used in the phylogenetic analysis in Figure 2) and their original copies (sequences used in the

phylogenetic analysis in Figure 1).

[2], The sequences used in the small data include the Brassicaceae-specific gene copies and the two parasitic plant gene copies (sequences used in the phylogenetic analysis in Figure 2).

[3], Sites undergone positive selection calculated by Bayes Empirical Bayes (BEB) analysis.

[4], not detected.
