# Supplementary material for: Root parasitic plant Orobanche aegyptiaca and shoot parasitic plant Cuscuta australis obtained Brassicaceae-specific strictosidine synthase-like genes by horizontal gene transfer
Source: BMC Plant Biol. 2014 Jan 13;14:19. doi: 10.1186/1471-2229-14-19 (PMC3893544; doi:10.1186/1471-2229-14-19)
Supplement: Additional file 8 — Results of positive selection using branch-site models. [file 1471-2229-14-19-S8.pdf]

Additional File 8. Results of positive selection using branch-site models.

Foreground	Model	LnL	Parameters	Positively selected sites[4]
Brassicaceae[1]	null	-13622.4689	$p_0 = 0.8100, p_1$=0.1372, p_{2a}=0.0452, $p_{2b} = 0.0077$<br>Background: $\omega_0 = 0.1361, \omega_1 = 1.0000, \omega_{2a} = 0.1361, \omega_{2b} = 1.0000$<br>Foreground: $\omega_0 = 0.1361, \omega_1 = 1.0000, \omega_{2a} = 1.0000, \omega_{2b} = 1.0000$	-
	alternative	-13622.4689	$p_0 = 0.8100, p_1 = 0.1372, p_{2a}$=0.0452, $p_{2b} = 0.0077$<br>Background: $\omega_0 = 0.1361, \omega_1 = 1.0000, \omega_{2a} = 0.1361, \omega_{2b} = 1.0000$<br>Foreground: $\omega_0 = 0.1361, \omega_1 = 1.0000, \omega_{2a} = 1.0000, \omega_{2b} = 1.0000$	NAN
Cuscuta australis[2]	null	-9568.8524	$p_0 = 0.7723, p_1 = 0.2116, p_{2a}$=0.0126, $p_{2b} = 0.0035$<br>Background: $\omega_0 = 0.1225, \omega_1 = 1.0000, \omega_{2a} = 0.1225, \omega_{2b} = 1.0000$<br>Foreground: $\omega_0 = 0.1225, \omega_1 = 1.0000, \omega_{2a} = 1.0000, \omega_{2b} = 1.0000$	-
	alternative	-9567.8055	$p_0 = 0.7772, p_1 = 0.2133, p_{2a}$=0.0075, $p_{2b} = 0.0021$<br>Background: $\omega_0 = 0.1219, \omega_1 = 1.0000, \omega_{2a} = 0.1219, \omega_{2b} = 1.0000$	NAN

			Foreground: $\omega_0 = 0.1219, \omega_1 = 1.0000, \omega_{2a} = 6.0725, \omega_{2b} = 6.0725$	
Orobanch aegyptiaca[3]	null	-9537.3278	$p_0 = 0.7502, p_1 = 0.2010, p_{2a} = 0.0311, p_{2b} = 0.0087$<br>Background: $\omega_0 = 0.1240, \omega_1 = 1.0000, \omega_{2a} = 0.1240, \omega_{2b} = 1.0000$<br>Foreground: $\omega_0 = 0.1240, \omega_1 = 1.0000, \omega_{2a} = 1.0000, \omega_{2b} = 1.0000$	-
	alternative	-9534.4507	$p_0 = 0.7750, p_1 = 0.2187, p_{2a} = 0.0050, p_{2b} = 0.0014$<br>Background: $\omega_0 = 0.1245, \omega_1 = 1.0000, \omega_{2a} = 0.1245, \omega_{2b} = 1.0000$<br>Foreground: $\omega_0 = 0.1245, \omega_1 = 1.0000, \omega_{2a} = 50.1481, \omega_{2b} = 50.1481$	181G, 267N*

[1], The large dataset, which contains the Brassicaceae-specific gene copies (sequences used in the phylogenetic analysis in Figure 2 and were set as the foreground) and their orginal gene copies (sequences used in the phylogenetic analysis of Figure 1 and were set as the background)

[2], The small dataset, which includes the Brassicaceae-specific gene copies [(sequences used in the phylogenetic analysis of Figure 2 and were set as the background) and the *CaSSL* gene in *Cuscuta australis* (set as foreground)].

[3], The small dataset, which includes the Brassicaceae specific gene copies [(sequences used in the phylogenetic analysis of Figure 2 and were set as the background) and the *OaSSL* gene in *Orobanche aegyptiaca* (set as foreground)].

[4], Tites undergone positive selection calculated by Bayes Empirical Bayes (BEB) analysis.

[*], The posterior probability is more than 0.95.
