# Supplementary material for: Root parasitic plant Orobanche aegyptiaca and shoot parasitic plant Cuscuta australis obtained Brassicaceae-specific strictosidine synthase-like genes by horizontal gene transfer
Source: BMC Plant Biol. 2014 Jan 13;14:19. doi: 10.1186/1471-2229-14-19 (PMC3893544; doi:10.1186/1471-2229-14-19)
Supplement: Additional file 9 — Sites undergone positive selection in the OaSSL gene of O. aegyptiaca. [file 1471-2229-14-19-S9.pdf]

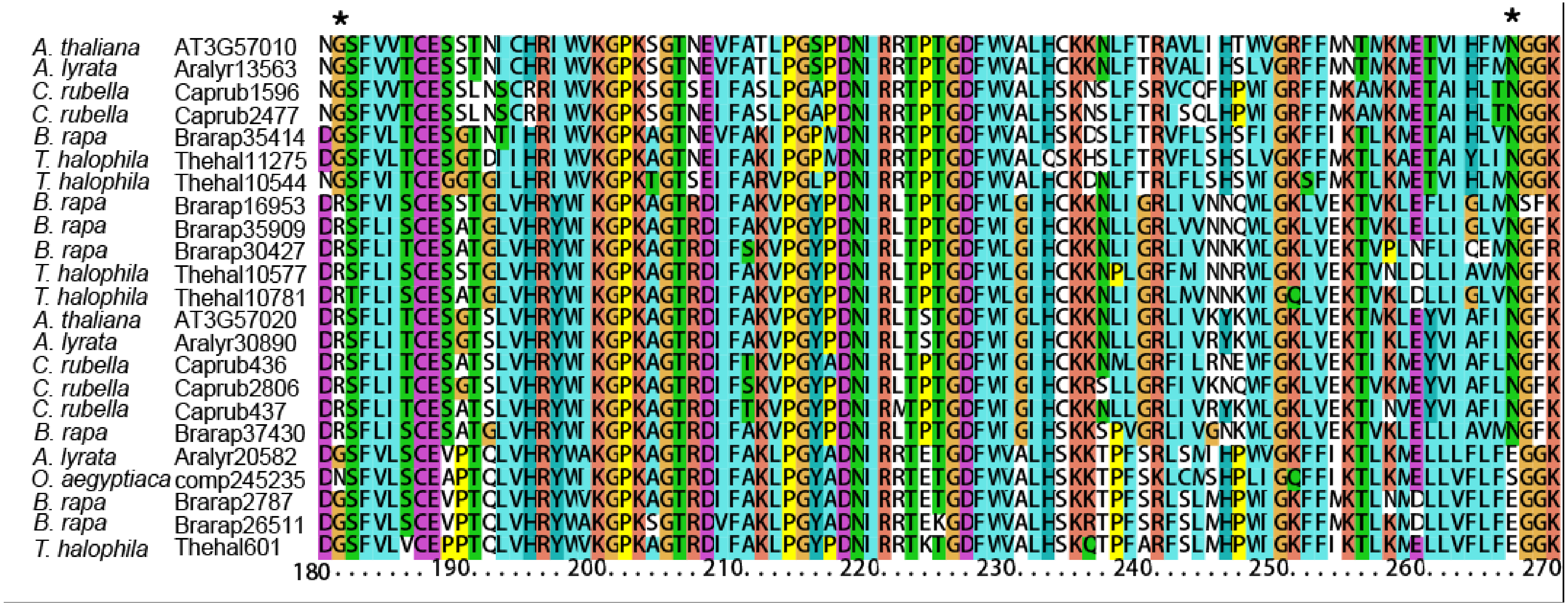

Additional File 9. Sites undergone positive selection in the *OaSSL* gene of *O. aegyptiaca*.

Two codon sites undergone positive selection, 181 and 267, are indicated by stars.
