# Supplementary material for: Root parasitic plant Orobanche aegyptiaca and shoot parasitic plant Cuscuta australis obtained Brassicaceae-specific strictosidine synthase-like genes by horizontal gene transfer
Source: BMC Plant Biol. 2014 Jan 13;14:19. doi: 10.1186/1471-2229-14-19 (PMC3893544; doi:10.1186/1471-2229-14-19)
Supplement: Additional file 5 — The AtSSL1 expression changes of A. thaliana upon different treatments calculated by GENEVESTIGATOR. [file 1471-2229-14-19-S5.pdf]

1880 of 1880 perturbations fulfilled the filter criteria

Filter values for ● AT2G41300

<< down-regulated | Log(2)-ratio | up-regulated >>

Arabidopsis thaliana (1880)	Log(2)-ratio	Fold-Change (no filter)	p-value (no filter)
H. schachtii / non-infected root samples	2.89	7.39	N/A
NAA study 3 (5d) / mock treated inflorescence stem internode starch sheath cell sam...	1.75	3.86	0.034
BL/H3BO3 (8d) / untreated cell culture samples	1.59	3.08	0.079
myb50 / Col-0	1.41	2.65	<0.001
shift to pH 4.6 / protoplasting / shift to pH 4.6 (24h)	1.40	2.67	0.022
iron deficiency / protoplasting / iron deficiency study 8 (24h)	1.19	2.27	0.003
shift to pH 4.6 (LZ3) / shift to pH 4.6 (LZ2)	1.15	2.22	0.007
BL/H3BO3 (6d) / untreated cell culture samples	1.14	2.26	0.151
rhd2-1 / Col-0	1.06	2.10	0.005
tcp2/tcp4ko / TCP4p::rTCP4:GFP	1.05	2.09	0.037
2,4-D + kinetin study 2 (ckh2-1) / 2,4-D study 2 (ckh2-1)	1.01	2.01	0.001
Bay-0 parent / Bur-0	0.95	1.89	0.033
ckh1-1 / Ler	0.94	1.93	0.007
BL/H3BO3 (10d) / untreated cell culture samples	0.93	1.90	0.110
bzr1-1Dxbri1-116 / bri1-116	0.91	1.88	0.005
Bay-0 parent / Sha	0.91	1.90	0.005
2,4-D + kinetin study 2 (Ler) / 2,4-D study 2 (Ler)	0.86	1.81	0.037
Bay-0 parent / Sha	0.84	1.81	0.006
circadian / ethanol / MG132 study 2 (Col-0) / circadian / ethanol / MG132 (Col-0)	0.82	1.75	0.002
Hh-0 / Bla-1/Hh-0	0.78	1.77	0.048
Bay-0 parent / C24	0.77	1.72	0.015
Bl-1 / Sha	0.76	1.69	0.018
brx / Sav-0	0.75	1.68	0.012
Bay-0 parent / Sha	0.75	1.67	0.020
2,4-D + kinetin (ckh1-1) / 2,4-D study 2 (ckh1-1)	0.73	1.66	0.013
C24_CAB2::LUC_CaMV35S::AEQ / toc1-1	0.72	1.66	0.054
upb1-1 / Col-0	0.71	1.64	0.026
Bl-1 / Bur-0	0.71	1.64	0.034
MeJa study 2 (3h) / mock treated seedlings (3h)	0.70	1.67	0.174
Bay-0 parent / Bur-0	0.69	1.63	0.039
2,4-D + kinetin study 2 (ckh1-1) / 2,4-D study 2 (ckh1-1)	0.69	1.62	0.024
OXUPB1-2 / Col-0	0.69	1.61	0.017
Bl-1 / Sha	0.68	1.61	0.003
brx / Sav-0	0.68	1.60	0.038
Bay-0 parent / Sha	0.65	1.58	0.010
HSP90(RNAi-A3) / Col-0	0.65	1.74	0.231
Bla-1 / Bla-1/Hh-0	0.64	1.55	0.003
Bl-1 / Sha	0.62	1.54	0.005
max4 / Col-0	0.59	1.50	0.006
salicylic acid study 5 (Van-0) / silwet L77 treated Van-0 leaf samples (28h)	0.58	1.49	0.071
BA (Col-0) / vehicle treated shoot samples (Col-0)	0.57	1.49	0.042
Bay-0 parent / Fei-0	0.56	1.48	0.022
M. incognita (early) / non-infected root samples (early)	0.55	1.46	<0.001
rga-delta17 / empty cassette	0.55	1.47	0.047
2,4-D + kinetin (ckh2-1) / 2,4-D study 2 (ckh2-1)	0.53	1.45	0.017
Bay-0 parent / Bur-0	0.51	1.43	0.050
ABA + salicylic acid (3h) / solvent treated cell samples (3h)	0.51	1.43	0.187
mkk1 / Col-0	0.50	1.44	0.071
rsl4-1 / Col-0	0.50	1.40	0.148
C24 / Bur-0	0.49	1.40	0.023
Bay-0 parent / Col-0	0.49	1.42	0.031
Bl-1 / Bur-0	0.49	1.36	0.153
lec1-1 / Ws-0	0.48	1.40	0.223
Ds13-2198-1 / n.s	0.47	1.39	0.144
MeJa study 3 (6h) / solvent treated cell culture samples (6h)	0.47	1.39	0.034
cngc19 / cngc1-3	0.47	1.42	0.301
circadian clock (Ca2+)cyt / nicotinamide (73h) / circadian clock (Ca2+)cyt study 7 (C2...	0.45	1.37	0.120
cov / Ler-0	0.44	1.36	0.061
BL/H3BO3 (4d) / untreated cell culture samples	0.44	1.34	0.240
P35S:rGRF1 / Col-0	0.44	1.35	0.002
35S-AtMYB44 / Col-0	0.44	1.37	0.272
35S::RSL4 / Col-0	0.43	1.34	0.244
rao1-1 / AOX1a:LUC	0.43	1.34	<0.001
P. syringae pv. tomato study 10 (DC3000) / mock inoculated leaf samples	0.42	1.37	0.191
circadian / ethanol / MG132 study 2 (Col-0) / circadian / MG132 study 2 (Col-0)	0.42	1.33	<0.001
tbf1 / Col-0	0.41	1.34	0.031
hypoxia study 3 (48h) / 21% O2 treated root samples (48h)	0.41	1.34	0.125
C24_CAB2::LUC_CaMV35S::AEQ / toc1-1	0.41	1.33	<0.001
sucrose / untreated seedlings	0.41	1.33	<0.001
Ws / Col-0	0.41	1.33	0.138
atgsnor1-3 / atgsnor1-1	0.40	1.32	N/A
C24_CAB2::LUC_CaMV35S::AEQ / toc1-1	0.40	1.32	0.077
ft-2 / Ler-0	0.40	1.33	0.031
M. incognita (late) / non-infected root samples (late)	0.40	1.32	0.097
lrx1_rol1-2 / Col-0	0.39	1.32	0.099
cpc-1 try-82 / Col-0	0.39	1.32	0.275
Bl-1 / C24	0.39	1.28	0.181
circadian / MG132 study 2 (Alc::TOC1) / circadian / MG132 (Alc::TOC1)	0.39	1.30	0.059
cry1 / Col-0	0.38	1.31	<0.001
atmyb44 / Col-0	0.38	1.30	0.207
NPA / mock treated seedlings	0.38	1.30	0.016
Bl-1 / Sha	0.38	1.30	0.029
fls2-17 / Ler-0	0.38	1.29	0.214
sucrose study 2 (AS-hyg) / dark grown ASN1::HPT2 seedlings	0.37	1.31	0.263
mpk6-3 / Col-0	0.37	1.31	0.267
rao1-2 / AOX1a:LUC	0.37	1.30	0.018
daminozide / mock treated seedlings	0.37	1.29	0.079
nudt7-1 sid2-1 eds1-2 / nudt7-1 sid2-1	0.37	1.29	0.032
IAA study 5 (hy5) / mock treated seedlings (hy5)	0.36	1.29	0.152
pad2 / coi1	0.36	1.27	0.069
Bay-0 parent / C24	0.35	1.26	0.238
callus formation study 3 (35d + 1d) / callus formation study 3 (7d + 1d)	0.35	1.27	0.013
mkk2 / Col-0	0.35	1.28	0.080
sulfur deficiency study 3 (LZ2) / sulfur deficiency study 3 (LZ1)	0.35	1.25	0.479
atgsnor1-3 / Col-0	0.34	1.27	N/A
tcp2/tcp4ko / TCP4p::wtTCP4:GFP	0.34	1.26	0.387
axr1-12 / Col-0	0.34	1.27	0.076
circadian clock (Ca2+)cyt / nicotinamide (81h) / circadian clock (Ca2+)cyt study 9 (C2...	0.34	1.27	0.053
ARF10 / Col-0	0.34	1.27	0.028
dark / 32°C (60-120min) / dark / 21°C (60-120min)	0.34	1.26	0.077
sid2 / coi1	0.33	1.25	0.061
mannitol study 2 (Col-0) / mock treated Col-0 guard cell samples	0.33	1.27	0.108
arf7:arf19 / Col-0	0.33	1.26	0.015
circadian clock (Ca2+)cyt / nicotinamide (61h) / circadian clock (Ca2+)cyt study 4 (C2...	0.33	1.26	0.112
Bay-0 parent / C24	0.33	1.27	0.126
zeatin study 3 (Col-0) / untreated whole plant samples (Col-0)	0.33	1.26	0.046
mock treated root samples (LZ2) / mock treated root samples (LZ1)	0.33	1.26	0.019
nudt7-1 sid2-1 eds1-2 / nudt7-1	0.33	1.26	0.029
C24_CAB2::LUC_CaMV35S::AEQ / toc1-1	0.33	1.25	0.053
D-biotin (bio4-1) / mock treated bio4-1 rosette leaf samples	0.32	1.25	0.016
16°C study 2 (pft1-1) / 23°C (pft1-1)	0.32	1.25	0.033
germination (12h) / desiccated seed samples	0.32	1.25	0.081
P. syringae pv. tomato study 15 (DC3000 avrRpm1) / P. syringae pv. tomato study 13 ...	0.32	1.25	0.120
light study 6 (csn4-1) / dark grown csn4-1 seedlings	0.31	1.28	0.400
pad2 / ein2	0.31	1.24	0.015
P35S:rGRF3 / Col-0	0.31	1.24	0.052
Bl-1 / C24	0.31	1.23	0.110
prohexadione / mock treated seedlings	0.31	1.24	0.050
BLS::TCP4 / Col-0	0.31	1.24	0.286
774 / C2S1	0.31	1.23	0.036
pad4 / coi1	0.31	1.23	0.115
cold study 2 (early) / untreated root samples (early)	0.31	1.25	0.021
salt study 2 (late) / untreated root samples (late)	0.30	1.23	0.012
arf7:arf19 / Col-0	0.29	1.22	0.072
atx1 / Ws	0.29	1.22	0.032
E. orontii (12h) / mock treated Col-0 leaf samples (12h)	0.29	1.22	0.120
sid2 / ein2	0.29	1.22	0.002
gun1-1 / Col-0	0.29	1.22	0.106
atcesA4 / atcesA4 het.	0.29	1.22	0.097
germination (48h) / desiccated seed samples	0.29	1.21	0.047
P. syringae pv. tomato study 14 (DC3000 avrRpm1) / P. syringae pv. tomato study 13 ...	0.28	1.22	0.031
Bay-0 parent / Col-0	0.28	1.23	0.166
OPDA study 2 (tga2-5-6) / solvent treated (tga2-5-6) seedlings	0.28	1.21	0.315
lec1-1 / Ws-0	0.28	1.21	0.039
aba1-1 / Ler-0	0.28	1.21	0.099
rpp4 / Col-0	0.28	1.20	0.336
slr-1 / Col-0	0.27	1.20	0.018
srk2cf / Col-0	0.27	1.21	0.022
170G-55-16/HGI2.1 / Cvi	0.27	1.21	0.015
Se-0 / Mir-0/Se-0	0.27	1.22	0.225
35S::HRE2 / Col-0	0.27	1.21	0.130
zeatin (3h) / mock treated seedlings (3h)	0.27	1.20	0.012
C24_CAB2::LUC_CaMV35S::AEQ / toc1-1	0.26	1.20	0.087
pad4 / ein2	0.26	1.20	0.046
ddm1-101 / Ws	0.26	1.18	0.251
Bl-1 / Col-0	0.26	1.19	0.216
ein3-1 eil1-1 / coi1-2	0.26	1.18	0.432
Ws-2 / Col-0	0.26	1.20	0.053
Tsu-1 / Kas-1	0.26	1.20	0.009
ACC (30min) / mock treated seedlings (30min)	0.26	1.20	0.222
low light / 21°C (300-360min) / moderate light / 21°C (300-360min)	0.26	1.19	0.006
dexamethasone study 3 (3d) / mock treated inflorescence tip samples (3d)	0.25	1.19	0.108
HIC / C24	0.25	1.18	0.393
callus formation (12h) / untreated root samples	0.25	1.17	0.534
lowR-FR study 2 (sav3-2) / continuous white light treated seedlings (sav3-2)	0.25	1.20	0.189
IAA study 9 (Bur-0) / untreated seedling samples (Bur-0)	0.25	1.16	0.422
nudt7-1 sid2-1 eds1-2 / Col-0	0.25	1.19	0.044
pifq / Col-0	0.25	1.18	0.094
sid2 / atgsnor1-1	0.25	1.19	0.013
transfection (HCRSV) / transfection (TCV)	0.25	1.20	0.323
csn4-1 / Col-0	0.25	1.24	0.443
dexamethasone study 2 / mock treated 35S::AP3-GR flower buds	0.25	1.17	0.252
syringolin study 2 / solvent treated leaf samples (syl_404_bc2)	0.25	1.19	0.107
gi11 / Ws	0.24	1.19	0.137
C24_CAB2::LUC_CaMV35S::AEQ / toc1-1	0.24	1.19	0.123
H. arabidopsidis study 2 (Col-0) / untreated seedling samples (Col-0)	0.24	1.18	0.402
dark study 2 (+DEX) / dark study 2 (-DEX)	0.24	1.18	0.045
Tsu-1 / Kas-1	0.24	1.19	0.231
circadian clock (Ca2+)cyt / nicotinamide (57h) / circadian clock (Ca2+)cyt study 3 (C2...	0.24	1.18	0.118
Bl-1 / Bur-0	0.24	1.18	0.165
germination (12h) / stratification (48h)	0.24	1.18	0.118
circadian / MG132 study 2 (Col-0) / circadian / MG132 (Col-0)	0.24	1.18	0.024
ms1-ttg / Ler-0	0.24	1.18	0.184
C24_CAB2::LUC_CaMV35S::AEQ / toc1-1	0.24	1.17	0.322
penta / Ler-0	0.24	1.18	0.049
tga2-1 tga3-1 tga5-1 tga6-1 / Col-0	0.24	1.18	0.026
4-thiazolidinone/acetic acid / solvent treated seedlings	0.24	1.15	0.299
callus formation study 3 (35d + 1d) / untreated hypocotyl samples (35d)	0.23	1.17	0.018
Ler-ttg / Ler-0	0.23	1.17	0.232
hai1-2 / Col-0	0.23	1.17	0.129
MeJa study 5 (gai) / untreated leaf disc samples (gai)	0.23	1.17	0.072
moderate light / 32°C (300-360min) / moderate light / 21°C (300-360min)	0.23	1.18	0.027
pad2 / Col-0	0.23	1.17	0.186
gh3.5-1D / Col-0	0.23	1.17	0.128
hypoxia study 11 (ate1-2 ate2-1) / mock treated seedling samples (ate1-2 ate2-1)	0.23	1.17	0.165
vip3 / n.s	0.23	1.18	0.234
sex1-3 / sex4-3	0.23	1.17	0.114
high light / 21°C (300-360min) / moderate light / 21°C (300-360min)	0.23	1.17	0.063
cpc-1 try-82 / Col-0	0.23	1.18	0.316
Bay-0 parent / Col-0	0.23	1.17	0.317
35S::amiRAP2.2-12 / Col-0	0.23	1.17	0.091
hypoxia study 5 (48h) / 21% O2 treated root samples (48h)	0.22	1.18	0.319
CAT2HP1 / Col-4	0.22	1.17	0.095
16°C study 2 (Col) / 23°C (Col)	0.22	1.16	0.141
co-2 / Ler-0	0.22	1.18	0.209
sex1-3 / Col-0	0.22	1.17	0.145
agl94 / Col-0	0.22	1.17	0.323
AtMYB30-ox-20A / Ws-4	0.22	1.17	0.469
16°C (det3) / untreated etiolated seedlings (det3)	0.22	1.16	0.238
hy5-215 / Col-0	0.22	1.17	0.296
35S::amiRAP2.12 / Col-0	0.22	1.17	0.134
16°C (Col-0) / untreated etiolated seedlings (Col-0)	0.22	1.16	0.109
Bl-1 / Bur-0	0.22	1.16	0.195
Bl-1 / C24	0.22	1.17	0.215
circadian clock study 3 (lhy-21cca11) / circadian clock study 4 (lhy-21cca11)	0.22	1.16	0.142
sid2 / atgsnor1-1	0.22	1.16	0.200
nudt7-1 sid2-1 eds1-2 / nudt7-1 eds1-2	0.21	1.16	0.067
cat2-1 / Col-0	0.21	1.16	0.180
P. syringae pv. tomato study 2 (DC3000 avrRpm1) / mock inoculated leaf samples (6...	0.21	1.17	0.182
CAT2HP1 / Col-4	0.21	1.17	0.445
sid2 / Col-0	0.21	1.15	0.181
ABA study 11 (24h) / solvent treated cell samples (24h)	0.21	1.16	0.124
iron deficiency study 7 (nas4x-1) / untreated leaf samples (nas4x-1)	0.21	1.13	0.473
sex1-3 / ptpks2	0.21	1.16	0.142
P. syringae pv. tomato study 9 (DC3118 Cor-hrpS) / mock inoculated leaf samples	0.21	1.16	0.290
Bay-0 parent / Bur-0	0.21	1.16	0.285
brx / Sav-0	0.21	1.15	0.033
germination (48h) / stratification (48h)	0.21	1.15	0.052
Bay-0 parent / C24	0.20	1.16	0.249
Fei-0 / Col-0	0.20	1.14	0.384
abi4-102 / Col-0	0.20	1.15	0.167
hy5 / Col-0	0.20	1.15	0.301
ara1OX / Col-0	0.20	1.15	0.513
penta / Ler-0	0.20	1.15	0.228
OE7a-1 / Col-0	0.20	1.15	0.124
drought study 11 (Col-0) / mock treated seedling samples (Col-0)	0.20	1.15	0.371
circadian clock (Ca2+)cyt / nicotinamide (93h) / circadian clock (Ca2+)cyt study 12 (C...	0.20	1.14	0.332
sucrose study 2 (cli186) / dark grown cli186 seedlings	0.20	1.15	0.444
KNO3 study 3 / (NH4)2SO4	0.20	1.15	0.204
high light study 3 (3h) / untreated leaf samples (CAT2HP1)	0.20	1.13	0.099
Pip3-3HA6H-1 / Nd-1	0.20	1.15	0.045
ABA (30min) / mock treated seedlings (30min)	0.20	1.14	0.051
long day (Col-0) / short day 2 (Col-0)	0.20	1.14	0.087
MeJa study 7 (24h) / solvent treated cell samples (24h)	0.20	1.14	0.095
npr1-1 / coi1	0.19	1.14	0.286
35S-AtMYB44 / Col-0	0.19	1.13	0.507
rotenone (3h) / solvent treated cell culture samples (3h)	0.19	1.13	0.103
S. malt: R3089 volatiles (12h) / mock treated seedling samples (12h)	0.19	1.16	0.160
NPA study 2 (7d) / mock treated stem samples (7d)	0.19	1.16	0.525
35S::amiR-white-1(MIR172a) / Col-0	0.19	1.18	0.548
35S::amiR-mads-1(MIR172a) / Col-0	0.19	1.14	0.195
cs26 / Col-0	0.19	1.15	0.285
KNO3/NH4NO3 study 2 (Col-0) / KNO3/NH4NO3 (Col-0)	0.19	1.14	0.078
etr1 / Col-0	0.19	1.14	0.054
sid2 / Col-0	0.19	1.15	0.185
AtMYB30-ox-20A / Ws-4	0.19	1.14	0.041
myb28 / Col-0	0.19	1.14	0.371
Bay-0 parent / Fei-0	0.19	1.15	0.371
AVG / mock treated seedlings	0.19	1.14	0.100
iron deficiency study 12 (Tsu-1) / mock treated Tsu-1 rosette samples	0.18	1.15	0.322
dark / 4°C (60-120min) / dark / 21°C (60-120min)	0.18	1.13	0.256
cordycepin (3h) / untreated cell samples	0.18	1.13	0.107
salicylic acid study 6 (Est) / silwet L77 treated Est leaf samples (52h)	0.18	1.13	0.244
pad4 / Col-0	0.18	1.13	0.300
tcp14 / Ws	0.18	1.12	0.456
heat study 6 (msh1-1 recA3-1) / untreated all aerial tissue samples (msh1-1 recA3-1)	0.18	1.13	0.165
nitrate starvation / untreated seedlings	0.18	1.13	0.246
lhy-21cca11-11 / Ws-0	0.18	1.13	0.069
elevated CO2 (48h) / ambient CO2 / ambient light treated developing leaf samples (4...	0.18	1.15	0.240
E. orontii (6h) / mock treated Col-0 leaf samples (6h)	0.18	1.14	0.107
HSP90(RNAi-A1) / Col-0	0.18	1.13	0.020
IAA study 7 (C24) / untreated seedling samples (C24)	0.18	1.13	0.271
gi11 / Ws	0.18	1.13	0.243
cat2-1 / Col-0	0.18	1.13	0.338
KNO3 study 4 (early) / KCl treated root samples (early)	0.18	1.13	0.253
IAA study 7 (Bur-0) / untreated seedling samples (Bur-0)	0.18	1.10	0.556
IAA study 3 / solvent treated seedlings	0.18	1.11	0.366
C24 / Bur-0	0.18	1.13	0.210
shift to pH 4.6 / FACS (pWOL::GFP) / root stele protoplast samples of mock treated p...	0.18	1.13	0.284
ced1 / Col-5	0.18	1.13	0.053
C24 / Bur-0	0.18	1.10	0.572
pif5-3 / Col-0	0.18	1.13	0.088
brassinazole (brz220) / mock treated seedlings	0.18	1.13	0.094
P. syringae pv. tomato (DC3000 hrcC-) / P. syringae pv. tomato (DC3000 avrRpm1)	0.18	1.12	0.221
hmi9-1 / Col-0	0.17	1.13	0.294
agb1-2 / Col-0	0.17	1.13	0.113
P. syringae pv. tomato study 15 (DC3000) / P. syringae pv. tomato study 13 (DC3000)	0.17	1.13	0.163
hypoxia study 5 (2h) / 21% O2 treated root samples (2h)	0.17	1.13	0.270
cat2-1 / Col-0	0.17	1.13	0.076
ckh1-1 / Ler	0.17	1.12	0.311
germination (24h) / desiccated seed samples	0.17	1.12	0.168
35S::HA::RAP2.12 / Col-0	0.17	1.13	0.179
dexamethasone / untreated seedlings	0.17	1.13	0.109
hsf1:hsf3 / Ws	0.17	1.13	0.390
C24_CAB2::LUC_CaMV35S::AEQ / toc1-1	0.17	1.12	0.140
2,4,6 T / mock treated seedlings	0.17	1.12	0.151
P. syringae pv. tomato study 4 (DC3000 hrpA-) / P. syringae pv. tomato study 4 (DC30...	0.17	1.12	0.085
temperature buffering study 2 (27°C) / 22°C grown seedling samples (GI11)	0.17	1.12	0.268
35S::ABF3-48 / control-48	0.17	1.13	0.302
hmi9-1 / Col-0	0.17	1.12	0.339
atgsnor1-3 / atgsnor1-1	0.17	1.12	0.265
white + far-red study 4 (pif5-3) / white study 4 (pif5-3)	0.17	1.12	0.041
2,4-D + trichostatin A (Ler) / 2,4-D study 2 (Ler)	0.16	1.12	0.375
circadian clock (Ca2+)cyt / nicotinamide (49h) / circadian clock (Ca2+)cyt (C24_LUC...	0.16	1.12	0.172
cold study 14 (24h) / untreated cell samples	0.16	1.12	0.279
low light / 21°C (60-120min) / moderate light / 21°C (60-120min)	0.16	1.12	0.174
pad2 / npr1-1	0.16	1.12	0.254
hfr1 / Col-0	0.16	1.12	0.172
C24_CAB2::LUC_CaMV35S::AEQ / toc1-1	0.16	1.11	0.622
elf3-7 / Col-0	0.16	1.11	0.334
BL (30min) / mock treated seedlings (30min)	0.16	1.12	0.124
KNO3 study 2 (root) / KCl treated root samples (Col-0)	0.16	1.11	0.481
P. syringae pv. tomato study 2 (DC3000 avrRpm1) / P. syringae pv. tomato study 2 (D...	0.16	1.13	0.306
tbf1 / Col-0	0.16	1.12	0.187
scm-2 / Col-0	0.16	1.11	0.265
iron deficiency study 2 (early) / mock treated root samples	0.16	1.12	0.357
PCIB / mock treated seedlings	0.16	1.12	0.318
estradiol study 3 (Estpro:FAMA) / mock treated Estpro:FAMA seedling samples (4h)	0.16	1.12	0.356
rotenone (12h) / solvent treated cell culture samples (12h)	0.16	1.11	0.172
MeJa study 2 (30min) / mock treated seedlings (30min)	0.15	1.11	0.073
hy5hyh / hyh	0.15	1.11	0.117
shift etiolated seedlings to light (late) / shift etiolated seedlings to light (early)	0.15	1.12	0.222
35S::MKS1 / mpk4	0.15	1.11	0.032
pGII-69-24 / Nd-1	0.15	1.11	0.132
nitrate (Col-0) / untreated root tissue samples (Col-0)	0.15	1.11	0.246
sfr2 / Col-0	0.15	1.11	0.265
ABA study 8 (gpa1-4) / solvent treated leaf samples (gpa1-4)	0.15	1.11	0.014
tcp13 / Ws	0.15	1.10	0.548
gun1:gun5 / Col-0	0.15	1.11	0.258
white + far-red study 2 (pifq) / white study 2 (pifq)	0.15	1.13	0.455
35S::HA::RAP2.12 / Col-0	0.15	1.11	0.031
npr1-1 / ein2	0.15	1.11	0.209
bzip1-1 / Col-0	0.15	1.11	0.245
ibuprofen / mock treated seedlings	0.15	1.11	0.188
P. syringae pv. tomato study 14 (DC3000 avrRpm1) / P. syringae pv. tomato study 14 ...	0.15	1.11	0.003
MeJa study 4 (Col-0) / mock treated plant samples (Col-0)	0.15	1.10	0.361
myb61 / Col-0	0.15	1.11	0.260
brassinazole (brz91) / mock treated seedlings	0.14	1.11	0.312
FLG22 study 2 (1h) / H2O treated Col-0 seedlings (1h)	0.14	1.11	0.375
transfection (HCRSV) / mock transfected protoplasts (Col-3)	0.14	1.11	0.600
ABA study 5 (srk2cf) / untreated plant samples (srk2cf)	0.14	1.10	0.215
KNO3 study 5 (1.5h) / N depletion study 2	0.14	1.11	0.010
arf7 / Col-0	0.14	1.10	0.418
P. syringae pv. tomato study 7 (pad4-5) / mock-inoculated leaf samples (pad4-5)	0.14	1.10	0.296
circadian clock (lhy-21cca1-11) / circadian clock study 4 (lhy-21cca1-11)	0.14	1.10	0.284
IAA study 6 / mock treated iaa1GR seedlings	0.14	1.09	0.551
mannitol study 2 (rgs1-2) / mock treated rgs1-2 guard cell samples	0.14	1.11	0.160
hen1-1 / Ler-0	0.14	1.09	0.460
MeJa study 3 (2h) / solvent treated cell culture samples (2h)	0.14	1.10	0.251
arf7 / Col-0	0.14	1.10	0.178
cry1 / Col-0	0.14	1.10	0.420
oas-a1.1 / Col-0	0.14	1.10	0.412
sid2 / npr1-1	0.14	1.10	0.245
hypoxia study 6 (Col-0) / untreated plant samples (Col-0)	0.14	1.10	0.148
ABA study 2 / untreated embryo endosperm samples	0.14	1.10	0.235
se-1 / Col-4	0.14	1.11	0.441
CCA1-34 / Col-0	0.14	1.10	0.416
circadian / ethanol / MG132 study 2 (Alc::TOC1) / circadian / ethanol / MG132 (Alc::TO...	0.14	1.09	0.367
GA4 study 5 (etr1-1) / solvent treated whole plant samples (etr1-1)	0.14	1.10	0.101
control-57 / control-48	0.14	1.10	0.176
osmotic study 4 (root) / mock treated Col-0 root samples	0.14	1.10	0.227
light study 5 (cli186) / dark grown cli186 seedlings	0.14	1.09	0.490
tcp13_tcp14 / Ws	0.14	1.09	0.578
atnob0-2 / Col-0	0.14	1.10	0.202
heat study 2 (ws) / untreated leaf samples (ws)	0.13	1.10	0.326
edr1 / Col-0	0.13	1.10	0.121
salicylic acid study 6 (Tsu-1) / silwet L77 treated Tsu-1 leaf samples (52h)	0.13	1.10	0.414
MG132 / mock treated seedlings	0.13	1.10	0.114
red study 2 (45h) / dark grown seedlings (pif1pif3pif4pif5)	0.13	1.09	0.226
drought study 4 (late) / untreated root samples (late)	0.13	1.10	0.182
fri:flc / FRI:FLC	0.13	1.09	0.301
salt study 5 (wer-1 myb23-1) / mock treated primary root tip samples (wer-1 myb23-1)	0.13	1.10	0.147
stratification (1h) / desiccated seed samples	0.13	1.11	0.596
rdr1-1 / Col-0	0.13	1.10	0.226
circadian clock study 3 (Ws-0) / circadian clock study 4 (Ws-0)	0.13	1.10	0.387
CO2 high / untreated leaf samples	0.13	1.13	0.733
35S::FAD3 / Col-0	0.13	1.09	0.181
35S::amiR-lfy-1(MIR172a) / Col-0	0.13	1.09	0.357
EF-Tu (elf18) (30min) / untreated whole plant samples (fls2-17)	0.13	1.09	0.018
cordycepin (1h) / untreated cell samples	0.13	1.09	0.162
lfy-12 / Col-0	0.13	1.09	0.341
H. arabidopsidis (4dpi + 6dpi) / mock treated cotyledon samples	0.13	1.09	0.486
mir-3TCP / Col-0	0.13	1.09	0.416
UV unfiltered max-310nm (1h) / seedlings irradiated with 327nm cut-off (1h)	0.13	1.10	0.363
abi4-102 vtc2-1 / vtc2-1	0.13	1.10	0.329
MPK6_1 / Col-0	0.13	1.09	0.300
KNO3 study 4 (late) / KCl treated root samples (late)	0.13	1.10	0.373
pye-1 / Col-0	0.13	1.09	0.171
P. syringae pv. tomato study 10 (DC3000 hrpA6C) / P. syringae pv. tomato study 10 (...	0.12	1.09	0.185
lrx1 / Col-0	0.12	1.08	0.404
sid2-2 / Col-0	0.12	1.09	0.127
CCA1-34 / Col-0	0.12	1.09	0.433
estradiol (6h) / DMSO treated plant samples (6h)	0.12	1.08	0.041
nas4x-1 / Col	0.12	1.09	0.364
P. syringae pv. tomato study 14 (DC3000 hrpA) / P. syringae pv. tomato study 14 (DC...	0.12	1.09	0.218
light study 4 (cli186) / dark grown cli186 seedlings	0.12	1.08	0.564
TuMV (zone 1) / leaf sap treated leaf samples	0.12	1.05	0.745
pif1-2 (dark) / Col-0 (dark)	0.12	1.09	0.191
sol2 / Utr	0.12	1.08	0.480
cold study 6 (Can) / 20°C/18°C treated rosette samples (Can)	0.12	1.09	0.380
ms1-ttg / Ler-0	0.12	1.09	0.512
shift NPA to naxillin (2h) / NPA study 3	0.12	1.08	0.335
sucrose study 3 (acn1-2) / untreated seedlings (acn1-2)	0.12	1.09	<0.001
heat study 8 (Col-0) / untreated Col-0 seedling samples	0.12	1.09	0.543
lec1-1 / Ws-0	0.12	1.11	0.726
low light / 4°C (140-200min) / low light / 21°C (140-200min)	0.12	1.09	0.182
FLG22 study 11 (1h) / FLG22 study 11 (0h)	0.12	1.07	0.524
UV filtered WG305 (6h) / seedlings irradiated with 327nm cut-off (6h)	0.12	1.10	0.106
salicylic acid study 5 (Est) / silwet L77 treated Est leaf samples (28h)	0.12	1.08	0.261
lhy-21cca11-11 / Ws-0	0.12	1.08	0.336
pif3-3 / Col-0	0.12	1.08	0.306
pmt4-1 / Col-0	0.12	1.09	0.398
KNO3 (Col-0) / untreated etiolated seedlings (Col-0)	0.11	1.08	0.313
shift NPA to NAA (2h) / NPA study 3	0.11	1.08	0.585
icl-2_ws / Ws-4	0.11	1.08	0.162
BL/H3BO3 (2d) / untreated cell culture samples	0.11	1.07	0.702
P. syringae pv. tomato study 9 (DC3118 Cor-) / mock inoculated leaf samples	0.11	1.08	0.178
cold study 4 (17°C) / 20°C treated rosette samples (6h)	0.11	1.08	0.409
gpa1 / Ws-2	0.11	1.08	0.202
KNO3/NH4NO3 study 2 (Col-0) / mock treated Col-0 whole plant samples (dark)	0.11	1.08	0.067
nudt7-1 eds1-2 / nudt7-1	0.11	1.08	0.095
pad4 / npr1-1	0.11	1.08	0.441
NAA + FLG22 (2h) / untreated leaf disc samples (Col-0)	0.11	1.08	0.079
germination (6h) / desiccated seed samples	0.11	1.08	0.435
P. syringae pv. syringae study 2 (Col-0) / P. syringae pv. syringae (Col-0)	0.11	1.08	0.303
Alc::TOC1 / Col-0	0.11	1.08	0.547
ABA study 6 (Col-0) / untreated plant samples (Col-0)	0.11	1.08	0.386
red study 4 (18h) / dark study 5 (18h)	0.11	1.08	0.024
circadian clock (Ca2+)cyt / nicotinamide (53h) / circadian clock (Ca2+)cyt study 2 (C2...	0.11	1.08	0.673
cold study 11 (ice1) / untreated whole plantlet samples (ice1)	0.11	1.07	0.542
X. campestris pv. campestris (AtMYB30-ox-20A) / untreated leaf samples (AtMYB30-o...	0.11	1.08	0.422
cat2-1 / Col-0	0.11	1.06	0.125
srk2cf / Col-0	0.11	1.08	0.320
GA3 (30min) / mock treated seedlings (30min)	0.11	1.08	0.359
Sha / Bur-0	0.11	1.05	0.711
zeatin (30min) / mock treated seedlings (30min)	0.11	1.08	0.174
cat2-1 / Col-0	0.11	1.07	0.447
TuMV (zone 0) / leaf sap treated leaf samples	0.11	1.11	0.791
agl65/66 / Col-0	0.11	1.08	0.110
B. graminis (Col-0) / non-infected rosette leaf samples	0.11	1.07	0.520
ABA study 7 (gpa1-4) / solvent treated guard cell samples (gpa1-4)	0.11	1.07	0.483
pif4-101 pif5-3 / Col-0	0.10	1.08	0.449
FRI:flc / FRI:FLC	0.10	1.07	0.410
npr1-1 sni1 brca2a / npr1-1 sni1	0.10	1.07	0.457
agl18 / Col-0	0.10	1.07	0.527
Mir-0 / Mir-0/Se-0	0.10	1.08	0.507
IAA study 7 (Fei-0) / untreated seedling samples (Fei-0)	0.10	1.08	0.424
iron deficiency study 9 (Kas-1) / mock treated root samples (Kas-1)	0.10	1.08	0.479
pkl-1 / Col-0	0.10	1.09	0.339
sid2-2 / Col-0	0.10	1.08	0.289
1135 / C2S1	0.10	1.07	0.442
P deficiency study 3 (Col-0) / untreated root samples (Col-0)	0.10	1.06	0.619
F. oxysporum (pft1-2) / mock treated pft1-2 leaf samples	0.10	1.08	0.477
KNO3/NH4NO3 study 2 (bzip1-1) / mock treated bzip1-1 whole plant samples (dark)	0.10	1.07	0.242
lhy-21cca11-11 / Ws-0	0.10	1.07	0.049
paclobutrazole / mock treated seedlings	0.10	1.07	0.405
circadian / ethanol / MG132 (Alc::TOC1) / circadian / MG132 (Alc::TOC1)	0.10	1.07	0.617
NAA study 3 (2d) / mock treated inflorescence stem internode starch sheath cell sam...	0.10	1.07	0.412
35S::MBF1c / Col-0	0.10	1.08	0.353
pif3-3 / Col-0	0.10	1.08	0.387
rao1-2 / AOX1a:LUC	0.10	1.07	0.293
sucrose study 3 (Col-7) / untreated seedlings (Col-7)	0.10	1.07	0.344
X. campestris pv. campestris study 2 (AtMYB30-ox-20A) / untreated leaf samples (At...	0.10	1.08	0.680
ccr1-1 / Col-0	0.10	1.07	0.469
hypoxia study 6 (ANAC102(KO-1)) / untreated plant samples (ANAC102(KO-1))	0.10	1.05	0.763
agl104 / Col-0	0.10	1.07	0.586
FLG22 study 4 (35S:AFB1) / untreated leaf disc samples (35S:AFB1)	0.10	1.07	0.406
pft1-1 / Col	0.10	1.07	0.284
lbd38 / lbd37-1	0.10	1.07	0.320
rao1-1 / AOX1a:LUC	0.10	1.07	0.371
npr1 / Col-0	0.10	1.05	0.769
Umkirch-1/Umkirch-3 / Uk-1	0.10	1.07	0.315
RBR depletion (RNAi; 12h) / H2O treated leaf samples (12h)	0.10	1.07	0.349
acn1-2 / Col-7	0.10	1.07	0.006
dark / 4°C (140-200min) / dark / 21°C (140-200min)	0.10	1.06	0.410
agb1-2 gpa1-4 / Col-0	0.10	1.07	0.373
salt (early) / untreated green tissue samples (early)	0.10	1.07	0.188
P. syringae pv. tomato study 4 (DC3000 hrpA-) / P. syringae pv. tomato study 4 (DC30...	0.10	1.07	0.403
toc1-1 / C24_CAB2::LUC_CaMV35S::AEQ	0.10	1.07	0.636
35S::AFB1 / Col-0	0.10	1.07	0.311
FLG22 study 7 (gai) / untreated leaf disc samples (gai)	0.10	1.07	0.574
triploidy / diploid seedlings	0.10	1.08	0.562
Fei-0 / Col-0	0.10	1.07	0.477
QK / Col-0	0.10	1.07	0.314
P. syringae pv. tomato study 9 (DC3118 Cor-hrpS) / P. syringae pv. tomato study 9 (D...	0.09	1.07	0.523
KCl treated root samples (early) / untreated root samples	0.09	1.08	0.613
IAA study 8 (C24) / untreated root tissue samples (C24)	0.09	1.06	0.530
nitrate (CHL1-5) / untreated root tissue samples (CHL1-5)	0.09	1.07	0.174
K+ deprivation (early) / non-deprived root samples (early)	0.09	1.07	0.044
C24 x Col-0 F1 hybrid / C24	0.09	1.05	0.577
germination (24h) / stratification (48h)	0.09	1.08	0.299
low light (48h) / ambient CO2 / ambient light treated developing leaf samples (48h)	0.09	1.07	0.309
elo3 / Ler-0	0.09	1.07	0.159
Bl-1 / Fei-0	0.09	1.07	0.428
fab1 / Col-0	0.09	1.06	N/A
shift to pH 4.6 / FACS (pWER::GFP) / root epidermis and lateral root cap protoplast s...	0.09	1.07	0.231
ckh2-1 / Ler	0.09	1.07	0.290
pifq / Col-0	0.09	1.08	0.672
white + far-red study 4 (pif4-101 pif5-3) / white study 4 (pif4-101 pif5-3)	0.09	1.07	0.534
iron deficiency (LZ1) / mock treated root samples (LZ1)	0.09	1.08	0.804
10°C / long day (Ler-0) / long day study 2 (Ler-0)	0.09	1.06	0.528
heat study 9 (photosynthesis 30% inhibited) / shift 22°C to optimal temperature	0.09	1.06	0.308
salt study 4 (Col(gl1)) / Hoagland solution watered Col(gl1) leaf samples	0.09	1.05	0.718
ft / Col-0	0.09	1.06	0.476
P deficiency study 4 (root) / mock treated Col-0 root samples	0.09	1.06	0.608
T8 / C2	0.09	1.06	0.462
[illegible]	[illegible]	[illegible]	[illegible]

Additional File 5. The *AtSSL1* expression changes of *A. thaliana* upon different treatments calculated by GENEVESTIGATOR.

The highlighted experiments were discussed in the main text.
